# Supplementary material for: Monocytes differentiate along two alternative pathways during sterile inflammation
Source: EMBO Rep. 2023 May 16;24(7):e56308. doi: 10.15252/embr.202256308 (PMC10328069; doi:10.15252/embr.202256308)
Supplement: Supplementary file 1 — Expanded View Figures PDF [file EMBR-24-e56308-s007.pdf]

Expanded View Figures

Figure EV1. Features of the scRNA-seq dataset.

A, B UMAP of integrated scRNAseq data from all time points. The donor origin of cells is shown. (B) Percentage of cells from each donor at different time points. Total number of cells is shown at the top of each bar.

C Number of genes (Features), counts (*n*Counts) and percentage of mitochondrial genes in scRNA-seq data for all time points and split by donor.

D Unsupervised clustering of all integrated data at resolution 0.2.

E Identification of possible contaminants. Enrichment of B cell, NK and T cell signatures is shown by clusters. Contaminating cells (2.8% of all cells) were excluded from subsequent analysis.

F–I Remapping of the data without contaminating cells. (F) UMAP of integrated scRNAseq data from all time points. The donor origin of cells is shown. (G) The time point origin of cells is shown. (H) Top differentially expressed genes between timepoints. (I) UMAP colored by RNA velocity pseudotime obtained with scVelo.

 © 2023 The Authors

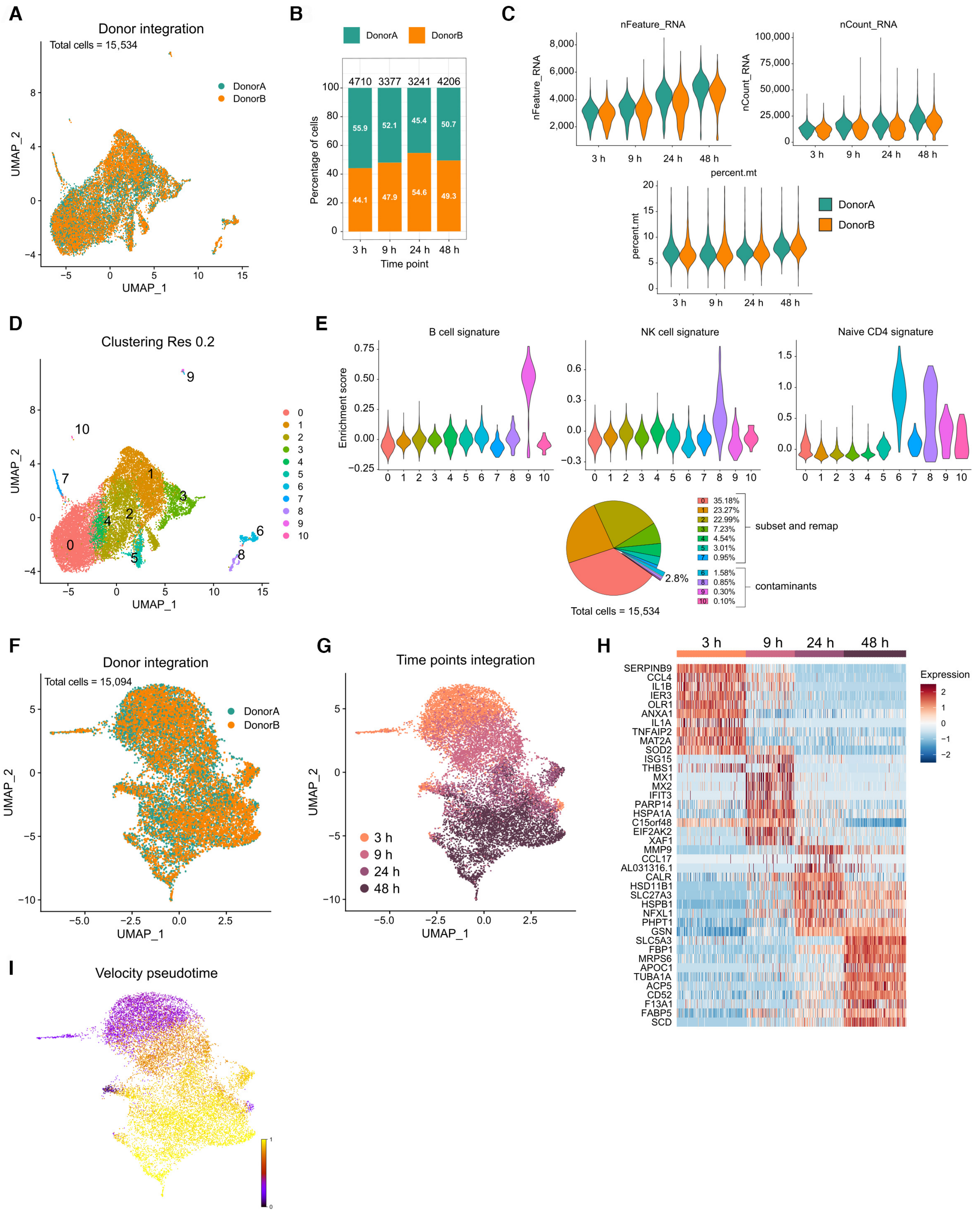
A
Donor integration
Total cells = 15,534
DonorA
DonorB
UMAP_1
UMAP_2
B
DonorA
DonorB
4710 3377 3241 4206
55.9 52.1 45.4 50.7
44.1 47.9 54.6 49.3
Percentage of cells
3 h 9 h 24 h 48 h
Time point
C
nFeature_RNA
nCount_RNA
percent.mt
DonorA
DonorB
D
Clustering Res 0.2
UMAP_1
UMAP_2
E
B cell signature
NK cell signature
Naive CD4 signature
Enrichment score
35.18%
23.27%
22.99%
7.23%
4.54%
3.01%
0.95%
subset and remap
1.58%
0.85%
0.30%
0.10%
contaminants
2.8%
Total cells = 15,534
F
Donor integration
Total cells = 15,094
DonorA
DonorB
UMAP_1
UMAP_2
G
Time points integration
3 h
9 h
24 h
48 h
UMAP_1
UMAP_2
H
3 h 9 h 24 h 48 h
SERPINB9
CCL4
IL1B
IER3
OLR1
ANXA1
IL1A
TNFAIP2
MAT2A
SOD2
ISG15
THBS1
MX1
MX2
IFIT3
PARP14
HSPA1A
C15orf48
EIF2AK2
XAF1
MMP9
CCL17
AL031316.1
CALR
HSD11B1
SLC27A3
HSPB1
NFXL1
PHPT1
GSN
SLC5A3
FBP1
MRPS6
APOC1
TUBA1A
ACP5
CD52
F13A1
FABP5
SCD
Expression
I
Velocity pseudotime

Figure EV1.

Figure EV2. Features of the peritonitis model.

A–C Peritonitis was induced by intra-peritoneal injection of thioglycolate. (A) CD45.2$^+$ Ly6C$^+$ monocytes were transferred into the inflamed peritoneum of CD45.1$^+$ mice. Strategy for the analysis of the time-course dataset. (B–C) Cells from the peritoneal lavage were analyzed by flow cytometry after 2 days. (B) Gating strategy is shown. (C) Expression of indicated markers. Representative results from one mouse are shown (n = 6 biological replicates). FMO = fluorescence-minus-one control.

D Purity of the bone marrow monocytes isolated with a magnetic kit. Representative results from one mouse are shown (n = 5 biological replicates). Percentage of monocytes, neutrophils and DC precursors in the purified cells (mean ± SD is shown).

© 2023 The Authors

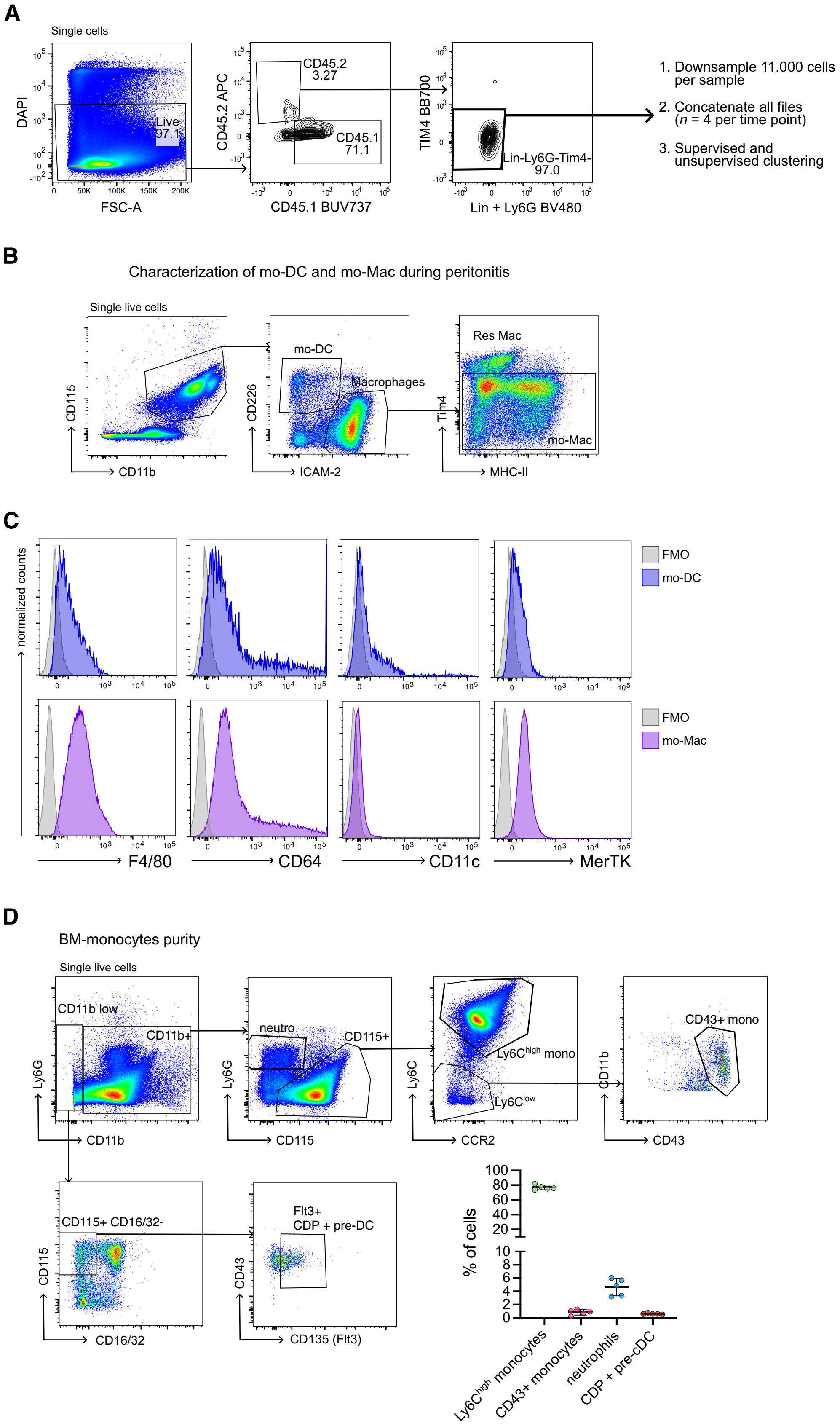
A
Single cells
DAPI
FSC-A
Live 97.1
CD45.2 APC
CD45.1 BUV737
CD45.2 3.27
CD45.1 71.1
TIM4 BB700
Lin + Ly6G BV480
Lin-Ly6G-Tim4- 97.0
1. Downsample 11.000 cells per sample
2. Concatenate all files (n = 4 per time point)
3. Supervised and unsupervised clustering
B
Characterization of mo-DC and mo-Mac during peritonitis
Single live cells
CD115
CD11b
mo-DC
Macrophages
CD226
ICAM-2
Res Mac
mo-Mac
Tim4
MHC-II
C
normalized counts
FMO
mo-DC
mo-Mac
F4/80
CD64
CD11c
MerTK
D
BM-monocytes purity
Single live cells
CD11b low
CD11b+
Ly6G
CD11b
neutro
CD115+
CD115
Ly6C^high mono
Ly6C^low
Ly6C
CCR2
CD43+ mono
CD11b
CD43
CD115+ CD16/32-
CD115
CD16/32
Flt3+
CDP + pre-DC
CD43
CD135 (Flt3)
% of cells
Ly6C^high monocytes
CD43+ monocytes
neutrophils
CDP + pre-cDC

Figure EV2.
