# Supplementary material for: Monocytes differentiate along two alternative pathways during sterile inflammation
Source: EMBO Rep. 2023 May 16;24(7):e56308. doi: 10.15252/embr.202256308 (PMC10328069; doi:10.15252/embr.202256308)
Supplement: Supplementary file 4 — PDF+ [file EMBR-24-e56308-s008.pdf]

Report

SOURCE DATA TRANSPARENT PROCESS OPEN ACCESS

EMBO reports

Monocytes differentiate along two alternative pathways during sterile inflammation

Javiera Villar, Léa Ouaknin, Adeline Cros & Elodie Segura*

Abstract

During inflammation, monocytes differentiate within tissues into macrophages (mo-Mac) or dendritic cells (mo-DC). Whether these two populations derive from alternative differentiation pathways or represent different stages along a continuum remains unclear. Here, we address this question using temporal single-cell RNA sequencing in an *in vitro* model, allowing the simultaneous differentiation of human mo-Mac and mo-DC. We find divergent differentiation paths, with a fate decision occurring within the first 24 h and confirm this result *in vivo* using a mouse model of sterile peritonitis. Using a computational approach, we identify candidate transcription factors potentially involved in monocyte fate commitment. We demonstrate that IRF1 is necessary for mo-Mac differentiation, independently of its role in regulating transcription of interferon-stimulated genes. In addition, we describe the transcription factors ZNF366 and MAFF as regulators of mo-DC development. Our results indicate that mo-Macs and mo-DCs represent two alternative cell fates requiring distinct transcription factors for their differentiation.

Keywords dendritic cells; differentiation; macrophages; monocytes
Subject Categories Chromatin, Transcription & Genomics; Development; Immunology
DOI 10.15252/embr.202256308 | Received 16 October 2022 | Revised 18 April 2023 | Accepted 2 May 2023 | Published online 16 May 2023
EMBO Reports (2023) 24: e56308

Introduction

Monocytes are key actors in the maintenance of tissue homeostasis and in inflammatory responses. After exiting the bone marrow, they circulate in the blood and can migrate to peripheral tissues where they rapidly differentiate into macrophages or dendritic cells (DC) (Jakubzick *et al*, 2017; Guilliams *et al*, 2018; Coillard & Segura, 2019). The factors orchestrating monocyte fate decision remain poorly understood. In particular, whether these two cellular identities represent alternative differentiation pathways or different stages along a continuum is controversial.

Monocyte-derived macrophages (mo-Mac) and DC (mo-DC) have been evidenced in multiple tissues during acute and chronic inflammation, in both mouse and human (Zigmond *et al*, 2012; Guilliams *et al*, 2018; Coillard & Segura, 2019). Mo-Mac and mo-DC are also present in the steady-state in skin, peritoneum, and Peyer's Patches (Tamoutounour *et al*, 2013; Bonnardel *et al*, 2015; Goudot *et al*, 2017). In other tissues at homeostasis, monocytes replenish the pool of macrophages over time, in particular in heart (Epelman *et al*, 2014; Molawi *et al*, 2014), intestine (Bain *et al*, 2014; Shaw *et al*, 2018) and pancreas (Calderon *et al*, 2015), but do not seem to differentiate into mo-DC in this context (Liu *et al*, 2019). Monocytes were initially proposed to be pre-committed to become mo-Mac or mo-DC (Menezes *et al*, 2016). Subsequently, we and others have shown that monocyte differentiation is not transcriptionally imprinted but can be oriented by external signals including cytokines, retinoic acid, and pathogen-derived products (Vento-Tormo *et al*, 2016; Goudot *et al*, 2017; Mildner *et al*, 2017; Sander *et al*, 2017; Devalaraja *et al*, 2020; Coillard *et al*, 2021).

Several molecular regulators involved in mo-DC development have been identified using *in vitro* models and include IRF4, aryl hydrocarbon receptor, BLIMP-1, NCOR2, miR-155, ETV3, and ETV6 (Briseño *et al*, 2016; Goudot *et al*, 2017; Sander *et al*, 2017; Coillard *et al*, 2021; Mendes *et al*, 2021; Villar *et al*, 2022). The role of IRF4, aryl hydrocarbon receptor was confirmed *in vivo* in genetically deficient mice in homeostatic conditions (Kim *et al*, 2016; Goudot *et al*, 2017). The orphan nuclear receptor NR4A3 was also shown to participate in mo-DC differentiation, acting downstream of IRF4, but only during inflammation *in vivo* (Salix *et al*, 2019). Similarly, ETV6 was found to control mo-DC differentiation *in vivo* during inflammation but not in steady-state (Villar *et al*, 2022). While the transcriptional regulation of macrophage identity in tissues is well characterized (Blériot *et al*, 2020; Guilliams *et al*, 2020), transcription factors driving the monocyte-to-macrophage program remain poorly characterized, besides our finding that MAFB is required for human mo-Mac differentiation (Goudot *et al*, 2017).

Based on the requirement for distinct transcription factors for their differentiation, we have proposed that mo-Mac and mo-DC constitute two distinct cell lineages stemming from monocytes (Goudot *et al*, 2017). Recently, single-cell analyses and trajectory reconstruction in a model of neuro-inflammation have suggested that mo-DC could represent an intermediate state of monocyte differentiation, with mo-Mac being the end stage

Institut Curie, PSL Research University, INSERM, U932, Paris, France
*Corresponding author. Tel: +33 156246720; E-mail: elodie.segura@curie.fr

© 2023 The Authors. Published under the terms of the CC BY 4.0 license.

(Amorim *et al*, 2022). The transcriptional control of these transitions was not explored. More work is therefore needed to better understand the molecular regulation of the monocyte differentiation program.

Here, we used temporal single-cell RNA sequencing (scRNA-seq) to analyze human monocyte differentiation trajectories in an *in vitro* system. We identify divergent differentiation paths toward mo-Mac versus mo-DC, with a fate decision occurring within the first 24 h. Using a fate-mapping approach in a model of sterile peritonitis in mouse, we confirm that monocytes also differentiate along two divergent trajectories *in vivo*. Using computational approaches, we identify candidate transcription factors involved in human monocyte differentiation. We further show that IRF1 is required for mo-Mac differentiation and that ZNF366 and MAFF are involved in mo-DC differentiation. Our results support a model in which mo-Mac and mo-DC derive from alternative differentiation pathways, controlled by distinct regulatory networks.

Results and Discussion

Temporal scRNA-seq analysis reveals early divergent paths of human monocyte differentiation

To dissect monocyte fate decision mechanisms, we used our previously established *in vitro* differentiation model, in which human monocytes are cultured with a cocktail of M-CSF, IL4, and TNF-α and differentiate in the same culture into mo-DC and mo-Mac resembling the ones found in clinical samples (Goudot *et al*, 2017). Of note, we have previously shown using scRNA-seq that monocytes used in these cultures do not contain contaminating DC precursors (Villar *et al*, 2022). To address whether monocytes followed a binary fate decision or differentiated along a continuum of states, we sought to reconstruct monocyte differentiation trajectories using single-cell transcriptomes. We profiled monocyte cultures from two individual donors by scRNA-seq using a droplet-based method and focused on early time points: 3, 9, 24, and 48 h after the start of the culture (Fig 1A). We integrated the different datasets using *STACAS* (Fig EV1A–C) (Andreatta & Carmona, 2021) and performed unsupervised clustering using a graph-based approach with the *Seurat* package (Hao *et al*, 2021) (Fig EV1D). We identified 3 clusters of contaminating B cells, NK cells, and T cells, that we excluded from subsequent analysis (Fig EV1E and F). Cells from both donors were distributed homogenously (Fig EV1F). Unsupervised analysis grouped cells according to time points (Figs 1A and EV1G). Analysis of differential gene expression between time points showed a temporal regulation of gene expression (Fig EV1H), validating the experimental set-up.

We identified 14 different clusters in the dataset, most of them with mixed sample origins (Fig 1B). Clusters 1 and 2 contained almost exclusively cells captured at 3 h, cluster 5 contained cells captured at 9 h, while clusters 11, 12, 13, and 14 displayed a majority of cells from the 48 h time point (Fig 1B). To decipher cluster identity, we analyzed the expression of the top differentially expressed genes between clusters (Fig 1C; Dataset EV1) and of canonical genes of monocytes (*S100A9*, *S100A8*, *VCAN*, *CD14*), mo-Mac (*MAFB*, *CXCL8*, *THBS1*, *APOE*) and mo-DC (*IRF4*, *PRDM1*, *CD1B*, *CD1A*; Fig 1D). Clusters 1, 2, 3 and 4 had the highest expression of monocyte genes. Consistent with this, clusters 1, 2, 3, 4, 5, and 6 were enriched for the monocyte signature compared to other clusters (Fig 1E). Macrophage genes were predominantly expressed in clusters 4, 5, 6, 13, and 14, including *APOC1*, sterol 27-hydroxylase (*CYP27A1*), fatty acid binding proteins *FABP4* and *FABP5*, and chemokine *CCL22*. DC genes were found in clusters 3, 7, 8, 11, and 12, including *CD1E* and *CST3*. Of note, cluster 10 contained cells from all time points and expressed genes consistent with a contaminating population of intermediate $CD14^+$ $CD16^+$ monocytes, including *FCGR3A* (encoding CD16) and *AIF1* (Villani *et al*, 2017). We have previously shown in this model that IRF4 and MAFB are necessary for mo-DC and mo-Mac differentiation respectively (Goudot *et al*, 2017). Their expression appeared largely exclusive, with *IRF4* expression highest in cluster 3 while *MAFB* was mainly expressed in clusters 1, 4, 5, and 6 (Fig 1E). These results suggest that clusters 1 and 2 correspond to undifferentiated monocytes, while cells from other clusters express either a DC- or a macrophage-oriented transcriptional program starting after 3–9 h.

To study differentiation trajectories, we analyzed RNA velocity using *scVelo* (Bergen *et al*, 2020). This method estimates the kinetics of mRNA splicing in single-cell transcriptomes, in order to reconstruct cellular dynamics. Inference of the velocity pseudotime using *scVelo* was able to reconstruct independently the temporal sequence of the dataset (Fig EV1I), confirming the robustness of this approach. RNA velocity projection indicated two main dynamics (Fig 1F). Clusters 1 and 2 showed a strong activity directed towards clusters 3 and 4, suggesting early transcriptional changes consecutive to fate decision. At later time points, two parallel differentiation trajectories were evidenced, with velocities from clusters 7 and 11 directed towards clusters 8 and 12 on one hand (DC path), and velocities from cluster 13 directed towards cluster 14 on the other hand (macrophage path). Collectively, these results suggest two divergent and alternative differentiation pathways (Fig 1G), with fate commitment occurring in the first 24 h after monocytes are exposed to differentiation cues.

Monocytes differentiate along two divergent pathways during inflammation in mice

To validate the physiological relevance of our findings, we sought to track *in vivo* monocyte differentiation in a tissue where mo-DC and mo-Mac coexist. To this end, we used a model of sterile peritonitis, induced by thioglycolate injection into the peritoneum. We have previously shown that mo-DC and macrophages can be distinguished in steady-state peritoneum by the markers CD226 and ICAM2, respectively (Goudot *et al*, 2017). To confirm that mo-DC and mo-Mac had a stable phenotype during peritonitis, we profiled the expression of DC and macrophage markers after thioglycolate injection. While both populations expressed CD64, only mo-DC expressed CD11c and mo-Mac displayed higher levels of F4/80 and MerTK (Fig EV2A). We adoptively transferred $CD45.2^+$ $Ly6C^+$ monocytes into the inflamed peritoneum of $CD45.1^+$ mice and analyzed by flow cytometry the $CD45.2^+$ monocyte-derived cells at different time points (Fig 2A), using a panel of surface markers for monocytes, mo-DC, and macrophages. We concatenated 11.000 downsampled $CD45.2^+$ cells for each time point and we performed

 © 2023 The Authors

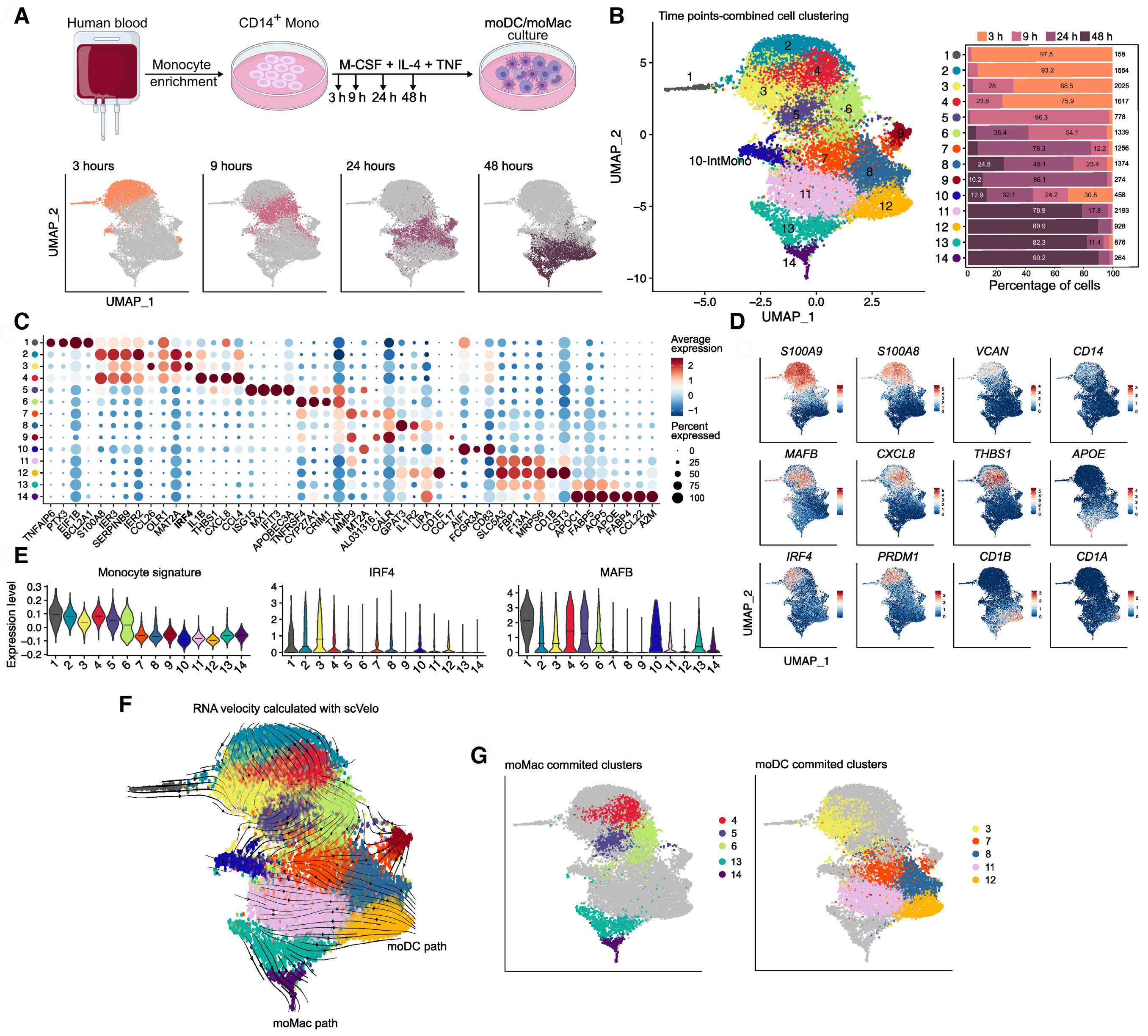

Figure 1. Temporal scRNA-seq analysis reveals early divergent paths of human monocyte differentiation.

CD14+ monocytes were cultured with M-CSF, IL-4, and TNFα and analyzed by scRNA-seq at different time points (*n* = 2 biological replicates).

A Experimental set-up. UMAP of single cells at each time point are shown.

B UMAP of integrated scRNAseq data from all time points. The sample origin of cells from each cluster is shown.

C Top differentially expressed genes between clusters.

D Expression patterns of monocytes (top row), macrophages (middle row), and DC (bottom row) markers.

E Normalized expression of monocyte gene signature, *IRF4* and *MAFB* in the different clusters.

F UMAP stream representation of RNA velocity calculated with scVelo.

G Identification of mo-mac and mo-DC committed clusters in the UMAP layout.

Source data are available online for this figure.

dimension reduction into a UMAP projection (Figs 2B and EV2B). Importantly, injected monocytes contained negligible quantities of DC precursors or Ly6C$^-$CD43$^+$ monocytes (Fig EV2C). Temporal analysis showed that the phenotype of CD45.2$^+$ monocyte-derived cells changed over time (Fig 2C–E), with monocyte markers Ly6C and CCR2 being down-regulated in the first 24 h and MHC II and CD115 being up-regulated within 24-48 h (Fig 2D). CD45.2$^+$ mo-DC (expressing CD11c, MHC II, and CD226) and mo-Mac (expressing ICAM2 and F4/80) emerged from 48 h (Fig 2D–G). To assess differentiation trajectories, we performed unsupervised clustering using

Phenograph, followed by lineage inference with *Slingshot* (Fig 2H–J) (Street *et al*, 2018). Monocytes (cluster 12, Fig 2H and I) were defined as the starting point of the pseudotime trajectory. This analysis identified several paths, with two distinct branching trajectories leading to mo-DC (clusters 8, 13, 18) or mo-mac (cluster 11) as terminal points (Fig 2J). These results suggest that *in vivo*, during inflammation, monocytes give rise to mo-DC or mo-Mac via two divergent differentiation pathways.

Identification of candidate transcription factors involved in monocyte differentiation

To identify transcriptional regulators of the early steps of monocyte differentiation, we used our human scRNA-seq dataset and analyzed differentially expressed genes between the clusters showing a commitment towards mo-DC versus mo-Mac, and extracted genes annotated as transcription factors. We focused on the comparison

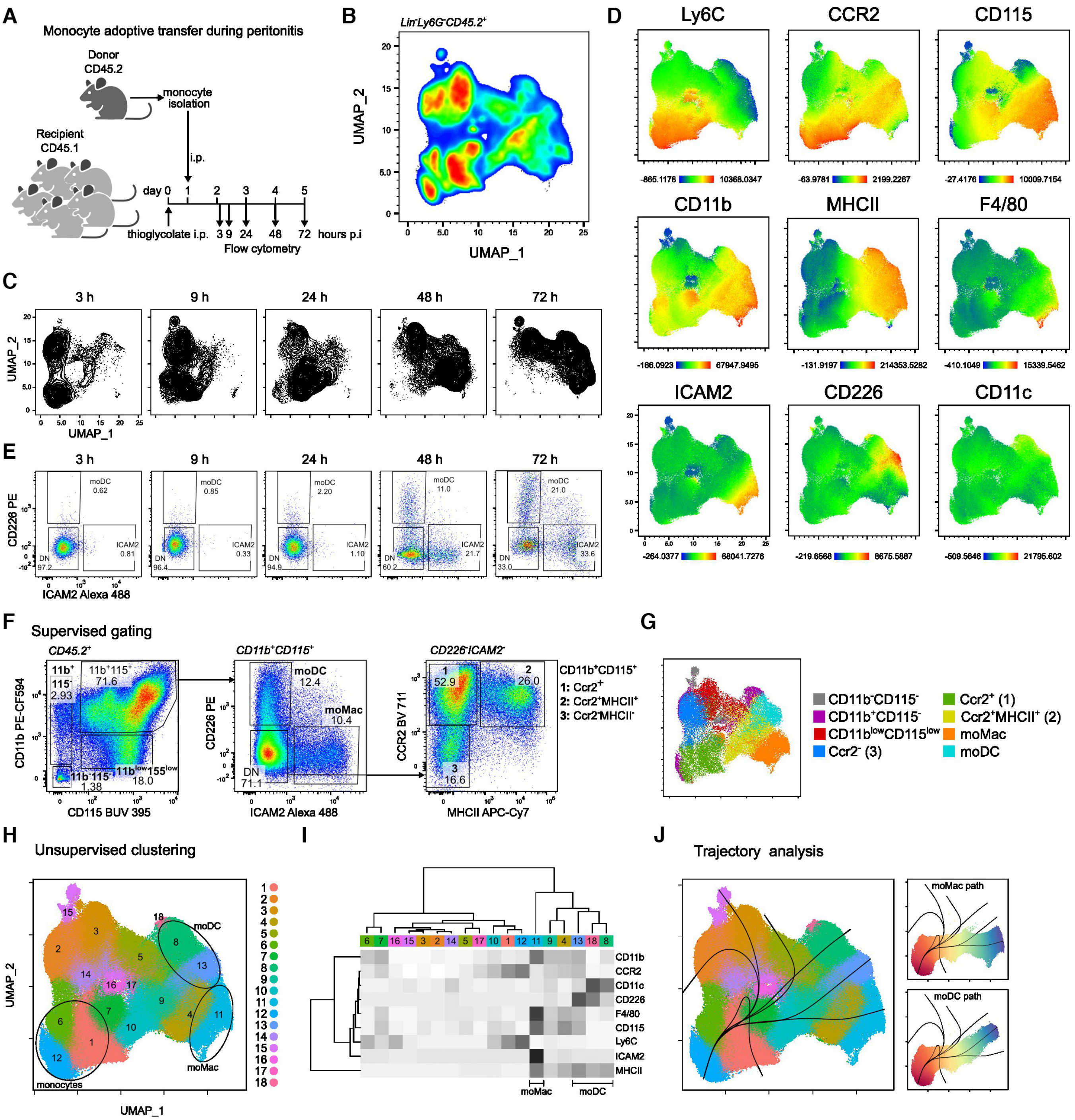

Figure 2.

 © 2023 The Authors

Figure 2. Monocytes differentiate along two parallel pathways during peritonitis *in vivo*.

A CD45.2^{+} monocytes were injected in CD45.1^{+} recipient mice previously injected with thioglycolate. Peritoneal lavage was analyzed at 3, 9, 24, 48, and 72 h post transfer (n = 4 biological replicates).
B UMAP displaying CD45.2^{+}Lineage^{-}Ly6G^{-}Tim4^{-} peritoneal cells analyzed by flow cytometry and concatenated from all time points.
C UMAP of cells from each time point.
D Expression of phenotypic markers used for UMAP projection.
E Supervised gating strategy for moDC and moMac at each time point (gated on live CD11b^{+} CD115^{+} cells).
F Supervised gating strategy to identify monocyte-derived cells.
G Distribution of manual gates in the UMAP projection.
H Unsupervised clustering using Phenograph.
I Heatmap of median marker expression values for each Phenograph cluster.
J Trajectory and pseudotime analysis using Slingshot. Cluster 12 (monocytes) was chosen as starting point. Trajectories from monocytes towards moDC or moMac are highlighted. Cells are colored by pseudotime.

between clusters 3 and 4 (Fig 3A), clusters 6 and 7 (Fig 3B), clusters 6 and 8 (Fig 3C), and clusters 12 and 14 (Fig 3D). To infer transcriptional activity, we also performed network analysis using *DoRoThEa* (Garcia-Alonso *et al*, 2019; Holland *et al*, 2020). Based on these analyses (Dataset EV2), we identified as candidate *IRF1*, which is more expressed in macrophage-engaged clusters 4 (Fig 3A) and 6 (Fig 3B and C) and shows high transcriptional activity in the clusters 5 and 6 (Fig 3E). Among differentially expressed transcription factors, we also selected as candidate *ZNF366*, which is more expressed in DC-committed clusters 3 (Fig 3A) and 8 (Fig 3C) and whose mouse ortholog is involved in classical DC1 (cDC1) terminal differentiation (Chopin *et al*, 2019; Shengbo *et al*, 2021). Finally, we selected *MAFF*, which is more expressed in macrophage-committed clusters 6 (Fig 3B) and 14 (Fig 3D) and heterodimerizes with the transcription factor NFE2L2 (Katsuoka & Yamamoto, 2016) which showed high predicted activity in clusters 5 and 6 (Fig 3E).

IRF1 controls mo-Mac differentiation independently of its role in regulating interferon-stimulated genes

To analyze the expression of IRF1 at the protein level, we performed Western Blot on freshly isolated monocytes and at different time points of culture (Fig 4A). IRF1 was expressed in monocytes and its expression was upregulated after 1 day of culture. To address the role of IRF1 in monocyte differentiation, we silenced its expression at the start of the culture using shRNA delivered through a lentivirus. We tested the impact of IRF1 deficiency on mo-Mac differentiation in a linear model (monocytes cultured with M-CSF alone in which only mo-Mac are produced), as well as our differentiation model with M-CSF, IL4, and TNF-α (Fig 4B–D). We used two different shRNA resulting in efficient silencing of IRF1 (Fig 4B). IRF1 silencing significantly impaired macrophage differentiation in both culture systems (Fig 4C and D), without affecting mo-DC

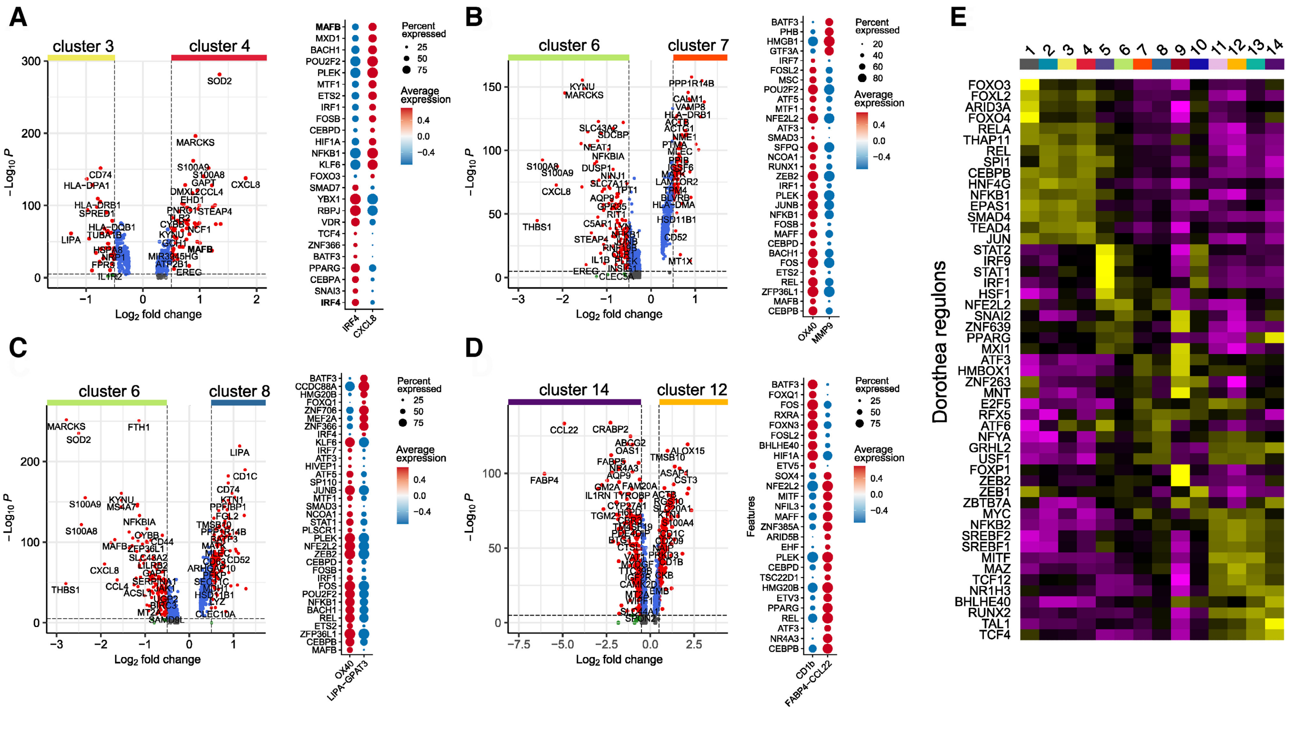

Figure 3. Identification of candidate transcription factors involved in monocyte differentiation.

A–D Differentially expressed genes between indicated clusters. Differentially expressed transcription factors are shown on the right.
E Transcription factor activity was calculated with Dorothea and Viper. Differentially activated regulons between clusters are shown.

© 2023 The Authors

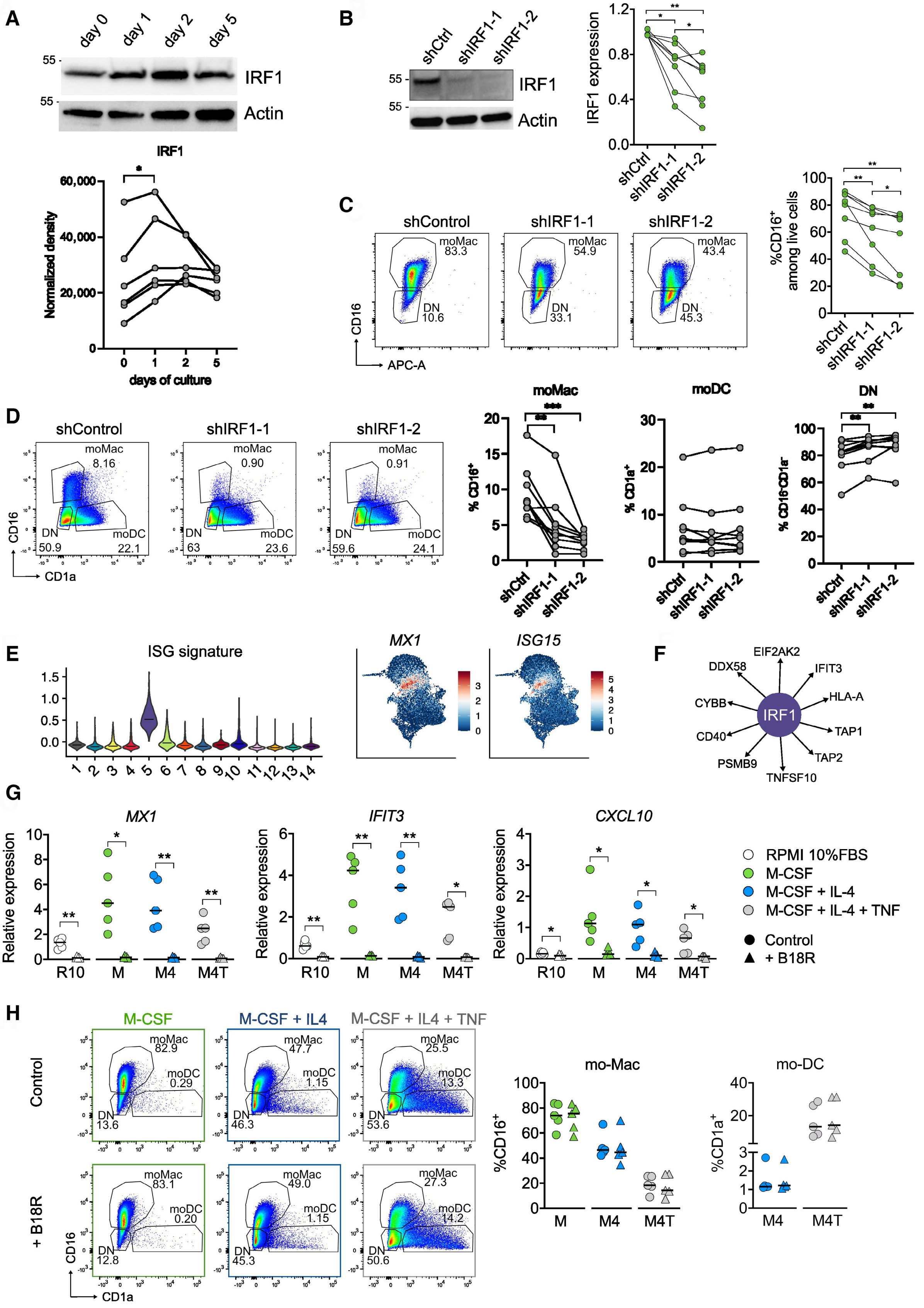

Figure 4.

 © 2023 The Authors

Figure 4. IRF1 controls mo-Mac differentiation independently of its regulation of interferon-stimulated genes.

A Monocytes were cultured with M-CSF, IL-4, and TNFα for 5 days. Protein quantification by Western Blot. Actin was used as loading control. Representative results are shown (*n* = 6 biological replicates). Quantification was performed by densitometry. Each symbol represents an individual donor. Paired one-way ANOVA.

B–D Monocytes were cultured with M-CSF (B, C) or M-CSF, IL-4, and TNFα (D) for 5 days. *IRF1* expression was silenced using a lentivirus containing shRNA. (B) Protein quantification by Western Blot after 5 days. Actin was used as loading control. Representative results are shown (*n* = 8 biological replicates). Quantification was performed by densitometry. Each symbol represents an individual donor. Paired one-way ANOVA. (C, D) Macrophage differentiation from monocytes was assessed by flow cytometry. One representative donor is shown (*n* = 8 for C and *n* = 9 for D). DN = double negative. Median is shown (*n* = 8–9 biological replicates in 3 independent experiments). Paired one-way ANOVA.

E Normalized expression of ISG signature in the different clusters. Pattern of expression for *MX1* and *ISG15*.

F Gene network from IRF1 regulon in cluster 5.

G, H Monocytes were cultured with RPMI medium (R10), or combinations of M-CSF, IL-4, and TNFα in the presence or absence of B18R (type I interferon inhibitor). (G) ISG expression after 9 h was analyzed by RT–qPCR. Each symbol represents an individual donor (*n* = 5 biological replicates). (H) Monocyte differentiation after 5 days. One representative donor is shown (*n* = 5 biological replicates). Proportion of mo-Mac and mo-DC after 5 days. Each symbol represents an individual donor (*n* = 5 biological replicates). Paired *t*-test.

Data information: For all panels: *P < 0.05, **P < 0.01, ***P < 0.001. Absence of star indicates 'not significant'.
Source data are available online for this figure.

differentiation (Fig 4D). These results validate a role for IRF1 in mo-Mac differentiation.

IRF1 is known to regulate the expression of interferon-stimulated genes (ISG) in macrophages (Kamijo *et al*, 1994; Langlais *et al*, 2016; Song *et al*, 2021; Rosain *et al*, 2023). Of note, cluster 5 showed high expression of ISG such as *MX1* and *ISG15* and was enriched for the ISG signature (Fig 4E). In addition, network analysis suggested that IRF1 controlled the expression of several ISG expressed in our dataset (Fig 4F). Therefore, we sought to address whether IRF1 may impact mo-Mac differentiation through the regulation of ISG expression. To confirm the expression of ISG during monocyte differentiation *in vitro*, we measured by RT–qPCR the expression of the ISG *MX1*, *IFIT3*, and *CXCL10* after 9 h of culture with different cytokine combinations (Fig 4G). We found that ISG expression were significantly induced upon exposure to M-CSF, with TNF-α decreasing ISG expression. To directly address the effect of ISG on monocyte differentiation, we used a soluble type I interferon receptor (B18R) which acts as an inhibitor of interferon signaling (Colamonici *et al*, 1995). We validated the inhibitory effect of B18R on the expression of *MX1*, *IFIT3*, and *CXCL10* (Fig 4G). However, we did not observe any impact of B18R on mo-Mac differentiation, whether using M-CSF alone, the combination of M-CSF and IL4, or the cocktail M-CSF, IL4, and TNF-α (Fig 4H). Taken together, these results suggest that IRF1 controls mo-Mac differentiation independently of its role in ISG expression.

IRF1 is known to be involved in myelopoiesis. *Irf1*-deficient mice display reduced granulocyte numbers, but have normal monocyte numbers in circulation with reduced expression of PU.1 (Testa *et al*, 2004). A role for IRF1 in the function and differentiation of osteoclasts, a bone-resident population of the monocyte lineage (Arai *et al*, 1999), was also proposed (Salem *et al*, 2014; Place *et al*, 2021). IRF1 was shown to modulate in human macrophages, but not in monocytes, the expression of ISG by controlling chromatin accessibility upon stimulation (Song *et al*, 2021). This finding together with our results suggests that the role of IRF1 in monocytes may be different from that observed in macrophages.

ZNF366/DC-SCRIPT is involved in mo-DC differentiation

We then examined the role of ZNF366 (also known as DC-SCRIPT) in monocyte differentiation. DC-SCRIPT was shown to be highly expressed in human DC, either derived *in vitro* or isolated from tissues (Triantis *et al*, 2006), and in murine cDC (Shengbo *et al*, 2021). *ZNF366* was highly expressed in DC-committed clusters 7, 8, and 12 (Fig 5A and B). The velocity of *ZNF366* was highest in the cluster 3, suggesting that the transcription of *ZNF366* was initiated in the first few hours of mo-DC fate decision (Fig 5A). To confirm this, we analyzed the protein expression at different time points using Western Blot (Fig 5C). ZNF366 was not detected in freshly isolated monocytes and its expression was induced upon culture. To directly address the role of ZNF366, we silenced its expression at the start of the culture using shRNA. We used two different shRNA with efficient down-regulation of ZNF366 (Fig 5D). Silencing of ZNF366 significantly decreased mo-DC differentiation without affecting mo-Mac (Fig 5E). Instead, we observed an increased proportion of $CD16^-CD1a^-$ cells, a population of undifferentiated cells (Goudot *et al*, 2017). These results indicate that ZNF366 is involved in mo-DC differentiation.

In mouse, DC-SCRIPT controls in cDC1 the production of IL-12, a cytokine expressed specifically by human mo-DC from clinical samples compared to mo-Mac (Tang-Huau *et al*, 2018). Understanding what gene programs are controlled by DC-SCRIPT during monocyte differentiation remains open for future investigation.

MAFF regulates mo-DC differentiation

Our transcriptomic data indicated that *MAFF* is more expressed in macrophage-committed clusters (Fig 5F). To analyze its expression at the protein level, we performed Western Blot at different time points (Fig 5G). MAFF was not detected in monocytes and was expressed upon culture. To test its role in monocyte differentiation, we silenced its expression as above and assessed the outcome of the monocyte culture. We used two different shRNA resulting in efficient decrease of MAFF expression (Fig 5H). Silencing of *MAFF* decreased mo-DC differentiation, without affecting mo-Mac proportions (Fig 5I). We also observed an increased proportion of undifferentiated $CD16^-CD1a^-$ cells.

MAFF belongs to the family of 'small' MAF proteins, which heterodimerize with other transcription factors including NF-E2-related factors, Bach proteins and c-Maf (Katsuoka & Yamamoto, 2016). MAFF has been reported to be a transcriptional repressor, but its function remains poorly understood (Blank, 2008; Katsuoka & Yamamoto, 2016). Our results show that MAFF is involved in mo-DC differentiation, although being enriched at the transcript level in

© 2023 The Authors

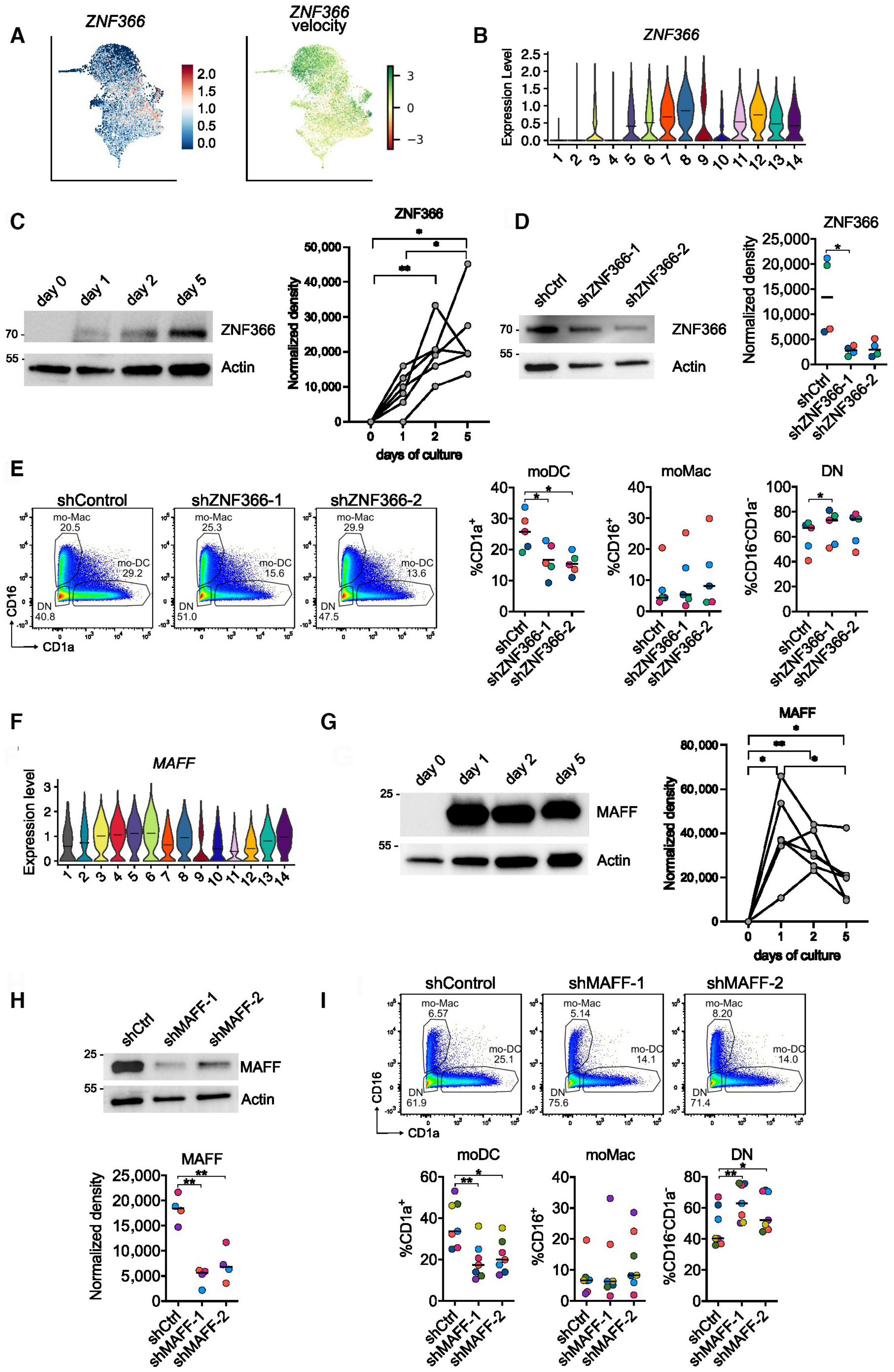

Figure 5.

© 2023 The Authors

Figure 5. ZNF366/DC-SCRIPT and MAFF are involved in mo-DC differentiation.

A *ZNF366* pattern of expression (left) and velocity (right).

B *ZNF366* expression across clusters.

C–G Monocytes were cultured with M-CSF, IL-4 and TNFα for 5 days. (C) Protein quantification by Western Blot. Actin was used as loading control. Representative results are shown ($n = 6$ biological replicates). Quantification was performed by densitometry. Each symbol represents an individual donor. Paired one-way ANOVA. (D–E) *ZNF366* expression was silenced using a lentivirus containing shRNA. (D) Protein quantification by Western Blot after 5 days. Actin was used as loading control. Representative results are shown ($n = 4$ biological replicates). Quantification was performed by densitometry. Each symbol represents an individual donor. Paired one-way ANOVA. (E) Mo-mac and mo-DC differentiation after 5 days was assessed by flow cytometry. One representative donor is shown ($n = 5$ biological replicates). DN = double negative. Proportion of mo-DC, mo-Mac and DN cells after 5 days. Median is shown ($n = 5$ biological replicates). Paired one-way ANOVA. (F) *MAFF* expression across clusters. (G) Protein quantification by Western Blot. Actin was used as loading control. Representative results are shown ($n = 6$ biological replicates). Quantification was performed by densitometry. Each symbol represents an individual donor. Paired one-way ANOVA.

H, I *MAFF* expression was silenced using a lentivirus containing shRNA. (H) Protein quantification by Western Blot after 5 days. Actin was used as loading control. Representative results are shown ($n = 4$ biological replicates). Quantification was performed by densitometry. Each symbol represents an individual donor. Paired one-way ANOVA. (I) Mo-mac and mo-DC differentiation after 5 days was assessed by flow cytometry. One representative donor is shown ($n = 7$ biological replicates). DN = double negative. Proportion of mo-DC, mo-Mac and DN cells after 5 days. Median is shown ($n = 7$ biological replicates). Paired one-way ANOVA.

Data information: $*P < 0.05$, $**P < 0.01$. Absence of star indicates ‘not significant’.
Source data are available online for this figure.

macrophage-oriented cells. This paradoxical finding suggests an indirect role for MAFF in the transcriptional regulation of monocyte differentiation that warrants further work.

In this work, we used temporal scRNA-seq analysis to reveal differentiation trajectories of human monocytes. In addition, we confirmed the existence of two parallel differentiation pathways *in vivo* in a mouse model of peritonitis. We identified IRF1, ZNF366/DC-SCRIPT and MAFF as molecular regulators of the early stages of monocyte differentiation. Our results contribute to the evidence supporting a model of divergent pathways leading to mo-DC versus mo-Mac differentiation. Recently, it was proposed in a model of neuro-inflammation that monocytes differentiate along a single path, with mo-DC being an intermediate and mo-Mac the end stage (Amorim *et al*, 2022). The discrepancy with our results may reflect context- or tissue-dependent phenomena. Because mo-DC and mo-Mac possess distinct functional properties, a better understanding of monocyte differentiation trajectories during pathogenic inflammation, and of their transcriptional control, will be essential for manipulating their fate for therapeutic purposes.

Materials and Methods

Mice

C57BL/6 mice ($CD45.2^+$) were purchased from Charles River (France). $CD45.1^+$ C57BL/6 mice were produced in-house. Mice were maintained under specific pathogen-free conditions at the animal facility of Institut Curie. Female mice were used at age 7–9 weeks. Sample size was not calculated *a priori*. No animal was excluded from analysis. Blinding was performed during outcome assessment. All animal procedures were in accordance with the guidelines and regulations of the French Veterinary Department and approved by the Institut Curie ethics committee (authorization APAFIS #25217-2020042522586261 v1).

Human samples

Buffy coats from healthy donors (both male and female donors) were obtained from Etablissement Français du Sang (Paris) in accordance with INSERM ethical guidelines. According to French Public Health Law (art L 1121-1-1, art L 1121-1-2), written consent and IRB approval are not required for human non-interventional studies.

Monocyte isolation and culture

Peripheral Blood Mononuclear Cells (PBMC) were prepared by centrifugation on a Ficoll gradient (Lymphoprep, StemCell). Blood CD14+ monocytes were isolated from healthy donors’ PBMC by positive selection using magnetic beads (Miltenyi). Monocytes (2×10^6 cells/ml) were cultured for indicated times in RPMI-Glutamax medium (GIBCO) supplemented with antibiotics (penicillin and streptomycin) and 10% Fetal Calf Serum in the presence or absence of 100 ng/ml M-CSF (Miltenyi), 5 ng/ml IL-4 (Miltenyi) and 5 ng/ml TNF-α (R&D Biotechne). Cytokines were added only at the start of the culture, and medium was not refreshed during the course of the culture. In some experiments, monocytes were cultured in the presence of 1 μg/ml of recombinant B18R (StemCell).

Single-cell RNA library preparation

For each time point, monocytes were detached from the plate and dead cells were removed with Dead Cell Removal Kit (Miltenyi) according to manufacturer’s instructions. Cells were barcoded per donor (Donor A and B) using TotalSeq™- anti-human Hashtag antibody (A0251, A02052, respectively; Biolegend) according to manufacturer’s instruction. Barcoded cells were counted and mixed in a 1:1 ratio. Finally, barcoded single-cell suspension was loaded into 10× Genomics Chromium (CA, USA). Libraries were prepared as per manufacturer’s protocol (Chromium Single Cell 3’ Reagent Kits v3 protocol) and sequenced on an Illumina NovaSeq sequencer according with 10× Genomics recommendations (paired-end reads) to a depth of approximately 50,000 reads per cell.

Single-cell RNA-sequencing analysis

Initial processing was done using *CellRanger* (v3.1.0). Starting from the filtered gene–cell count matrix produced by *CellRanger*, we proceeded with the *Seurat* v4.0 workflow (Hao *et al*, 2021) for demultiplex cells based on hashtag barcode and quality control (QC) processing. Briefly, we filtered out cells with mitochondrial genes < 20%, as well as genes expressed in less than 3 cells.

Hashtag demultiplexing was performed with the function HTODemux() and positive.quantile = 0.99. Integration of the 8 samples (2 donors, 4 time points) was subsequently performed with the *STACAS* workflow (Andreatta & Carmona, 2021). Briefly, normalization and identification of variable features was performed with standard *Seurat* pipelines and then anchors were identified with FindAnchors.STACAS (. . ., dims = 1:30, anchor.features = 5,000) and filter with FilterAnchors.STACAS (. . ., dist.thr = 0.8). The order for integration was calculated with SampleTree.STACAS(). Finally, integration was performed with the filtered anchors using the function IntegrateData(). Downstream analysis, graph-based clustering, visualization and differential gene expression analyses of the scRNA-seq data were performed using *Seurat* v4.0. For clustering analysis, FindNeighbors() and FindClusters() functions of the *Seurat* package were used with the first 50 significant PCs and a resolution of 1.3, respectively. For signatures enrichment, we used the function AddModuleScore(). For identification of DEGs, we used the FindMarkers or FindAllMarkers function (test.use = 't', logfc.threshold = log[0.25]) based on normalized data. DEGs with adjusted P values of > 0.05 were filtered out. Data has been deposited in GEO (accession number GSE218483).

RNA velocity

Annotations of unspliced/spliced reads were obtained using *velocyto CLI* with default parameters (La Manno *et al*, 2018). Next, unspliced counts were merged with the preprocessed, normalized, integrated and annotated spliced count matrix (*Seurat* v4.0) and the RNA velocity analysis was performed using *scVelo* (v0.2.4) workflow (Bergen *et al*, 2020). Briefly, we computed moments for velocity estimation and then the stochastic model to learn the transcriptional dynamics of splicing kinetics, transcriptional state, and velocity pseudo-time across the complete dataset.

Monocyte adoptive transfer

Monocytes were isolated from the pooled bone marrows of 3 individual mice using the EasySep Mouse Monocyte Isolation Kit (Stemcell) according to manufacturer's instructions. 1 million monocytes were injected intra-peritonally into CD45.1 C57BL/6 mice which had been injected 18 h before with 1 ml of 3.8% brewer's thioglycolate medium (Sigma). Peritoneal lavage was analyzed by flow cytometry at indicated time points after monocyte injection. Peritoneal lavage was recovered by intraperitoneal injection of 5 ml of PBS.

Flow cytometry

Human cells were stained in PBS containing 0.5% human AB serum and 2 mM EDTA for 30–45 min on ice with APC anti-CD1a (Biolegend, clone HI149) and FITC anti-CD16 (Biolegend, clone 3G8). For experiments involving shRNA, cells were stained with fixable Aqua Live/Dead (ThermoFisher) for 10 min at 4°C, and fixed prior to acquisition with fixation buffer from eBioscience Intracellular Fixation and Permeabilization kit (ThermoFisher). Otherwise, DAPI (Fischer Scientific, 100 ng/ml) was added immediately prior to acquisition. Cells were acquired on a FacsVerse instrument (BD Biosciences).

Mouse cells were stained in PBS containing BSA 0.5% and 2 mM EDTA for 30–45 min on ice. Antibodies used were anti-CD115 BUV 395 (BD Bioscience, clone AFS98), anti-CD172a BUV737 (BD Biosciences, clone P84), anti-Ly6G BV510 (Biolegend, clone 1A8), anti-CD19 BV480 (BD Bioscience clone 1D3), anti-TCRβ BV480 (BD Bioscience, clone H57-597), anti-NK1.1 BV480 (BD Bioscience, clone PK136), anti-SiglecF BV480 (BD Bioscience, clone E50-2440), anti-Ly6G BV480 (Biolegend, clone 1A8), anti-Ly6G BV605 (Biolegend, clone 1A8), anti-MHC II BV650 (Biolegend, clone M5/114.15.2), anti-CCR2 BV711 (BD Bioscience, clone 475,301), anti-CD11c BV786 (Biolegend, clone N418), anti-Ly6C BV786 (Biolegend, clone HK1.4), anti-Ly6C FITC (Biolegend, clone HK1.4), anti-CD45.2 APC (Biolegend, clone 104), anti-CD26 PE (Biolegend, clone H194-112), anti-CD226 PE (Biolegend, clone 10E5), anti-CD11b PE da594 (BD Bioscience, clone M1/70), anti-CD117 PE dazzle594 (Biolegend, clone 2B8), anti-CD11b PE (BD Biosciences, clone M1/70), anti-CD11b PerCPCy5.5 (BD Biosciences, clone M1/70), anti-F4/80 PECy7 (Biolegend, clone BM8), anti-CD16/32 PECy7 (Biolegend, clone 93), anti-CD64 PeCy7 (BD Bioscience, clone 10.1), anti-CD11c PeCy7 (BD Bioscience, clone N418), anti-MerTK PECy7 (Biolegend, clone 2B10C42), anti-ESAM APC (Biolegend, clone 1G8/ESAM), anti-CD115 APC (BD Bioscience, clone AFS98), anti-CD115 APC-Fire750 (BD Bioscience, clone AFS98), anti-TIM4 BB700 (Biolegend, clone RMT4-54), anti-CD43 BB700 (BD Bioscience, clone S7), anti-CD135 APC (Biolegend, clone A2F10), anti-Ly6C Alexa 700 (Biolegend, clone HK1.4), anti-MHC II APC Cy7 (Biolegend, clone M5/114.15.2) and anti-ICAM2 FITC (Biolegend, clone 3C4).

Data were analyzed with *FlowJo* (FlowJo LLC). Dimension reduction and clustering were performed in *FlowJo* using UMAP and Phenograph (Levine *et al*, 2015) modules, respectively, with default settings. For trajectory analysis, flow cytometry data was exported to R to perform *Slingshot* pseudo-time analysis (Street *et al*, 2018).

shRNA interference

shRNA (all from Sigma) against IRF1 (sh1: NM_002198-TRCN0000014669, sh2: NM_002198-TRCN0000014672), MAFF (NM_012323, sh1: TRCN0000016448, sh2: TRCN0000016449), ZNF266 (NM_152625, sh1: TRCN0000020134, sh2: TRCN0000020135), or nontargeting control shRNA (MISSION shRNA SHC002) were in the LKO.1-puro vector (MISSION® Sigma). Viral particles were produced by transfection of 293FT cells in 6-well plates with 3 mg DNA and 8 μL TransIT-293 (Mirus Bio) per well: for VSV-G pseudotyped SIVmac VLPs, 0.4 mg CMV-VSVG and 2.6 mg pSIV3+; for shRNA vectors, 0.4 mg CMV-VSV-G, 1 mg psPAX2 and 1.6 mg LKO1puro-derived shRNA vector. One day after 293FT cells transfection, medium was replaced by fresh culture medium. Viral supernatants were harvested 1 day later and filtered through 0.45 μm filters. Freshly isolated CD14+ monocytes were infected with viral particles containing the control vector or individual shRNA vectors, and cultured as above. Puromycin (InvivoGen) was added 2 days later (2 mg/ml). At day 5, cells were harvested for analysis.

qPCR

Cells were harvested and lysed in RLT buffer (QIAGEN). RNA extraction was carried out using the RNAeasy micro kit (QIAGEN) according to manufacturer's instructions. Total RNA was retro-transcribed using

© 2023 The Authors

the superscript II polymerase (Invitrogen), in combination with random hexamers, oligo dT, and dNTPs (Promega). Transcripts were quantified by real-time PCR on a 480 LightCycler instrument (Roche). Reactions were carried out in 10 µl, using a master mix (Eurogentec), with the following Taqman Assays primers (Merk), for human samples: B2M (Hs99999907_m1), RPL34 (Hs00241560_m1), HPRT1 (Hs02800695_m1), MX1 (Hs00895608_m1), IFIT3 (Hs00155468_m1), CXCL10 (Hs00895608_m1). The second derivative method was used to determine each Cp and the expression of genes of interest relative to the housekeeping genes (B2M, HPRT, RPL34) was quantified.

Immuno blot

Cells were lysed in RIPA buffer (Thermo Scientific) supplemented with complete Mini EDTA-free protease inhibitor cocktail (Roche), at 1×10^6 cells in 100 µl of lysis buffer. Post-nuclear lysates were resolved by SDS–PAGE using 4–15% BisTris NuPAGE gels (Invitrogen) and proteins were transferred to membranes (Immunoblot PVDF membranes, Bio-Rad). Membranes were stained with primary antibodies against IRF1 (Cell Signaling, clone D5E4), ZNF366 (Novus Biologicals, polyclonal, reference AF4707), MAFF (Novus Biologicals, polyclonal, reference AF3917), or actin (Millipore, clone C4), followed by HRP-conjugated secondary antibodies (Jackson Immunoresearch). Some membranes were incubated with "Re-blot Plus" buffer (Millipore).

Statistical analysis

Statistical tests were performed using *Prism* v9 (GraphPad Software). Statistical details for each experiment can be found in the corresponding figure legend. *N* corresponds to the number of biological replicates.

Data availability

The sequencing data from this publication have been deposited to the GEO database (GSM6746000; https://www.ncbi.nlm.nih.gov/geo/query/acc.cgi?acc=GSM6746000) and assigned the identifier GSE218483 (https://www.ncbi.nlm.nih.gov/geo/query/acc.cgi?acc=GSE218483).

The flow cytometry data from this publication have been deposited to the Flow repository database (http://flowrepository.org/id/FR-FCM-Z678 and assigned the identifier FR-FCM-Z678).

Expanded View for this article is available online.

Acknowledgements
This work was funded by INSERM, Institut Curie (CIC IGR-Curie 1428), the Agence Nationale de la Recherche (ANR-10-LABX-0043, ANR-10-IDEX-0001-02 PSL, and ANR-17-CE15-0011-01). JV is a fellow of the IC-3i PhD program supported by the ERC Horizon 2020-Marie Sklodowska-Curie Actions (grant #666003) and of the Fondation pour la Recherche Médicale (FDT202106013025). The authors wish to thank the Next Generation Sequencing Platform, the *In vivo* experiments Platform, and the Flow Cytometry Core of Institut Curie. The authors wish to thank J.Waterfall and L.Périé for helpful advice, and S.Heurtebise-Chrétien for managing the CD45.1 mouse colony.

Author contributions
Javiera Villar: Conceptualization; formal analysis; investigation; methodology; writing – original draft; writing – review and editing. **Léa Ouaknin:** Investigation; writing – review and editing. **Adeline Cros:** Investigation; writing – review and editing. **Elodie Segura:** Conceptualization; formal analysis; supervision; investigation; visualization; methodology; writing – original draft; writing – review and editing.

Disclosure and competing interests statement
The authors declare that they have no conflict of interest.

References

Amorim A, De Feo D, Friebel E, Ingelfinger F, Anderfuhren CD, Krishnarajah S, Andreadou M, Welsh CA, Liu Z, Ginhoux F *et al* (2022) IFNγ and GM-CSF control complementary differentiation programs in the monocyte-to-phagocyte transition during neuroinflammation. *Nat Immunol* 23: 217–228

Andreatta M, Carmona SJ (2021) STACAS: Sub-Type Anchor Correction for Alignment in Seurat to integrate single-cell RNA-seq data. *Bioinformatics* 37: 882–884

Arai F, Miyamoto T, Ohneda O, Inada T, Sudo T, Brasel K, Miyata T, Anderson DM, Suda T (1999) Commitment and differentiation of osteoclast precursor cells by the sequential expression of c-Fms and receptor activator of nuclear factor κb (rank) receptors. *J Exp Med* 190: 1741–1754

Bain CC, Bravo-Blas A, Scott CL, Gomez Perdiguero E, Geissmann F, Henri S, Malissen B, Osborne LC, Artis D, Mowat AM (2014) Constant replenishment from circulating monocytes maintains the macrophage pool in the intestine of adult mice. *Nat Immunol* 15: 929–937

Bergen V, Lange M, Peidli S, Wolf FA, Theis FJ (2020) Generalizing RNA velocity to transient cell states through dynamical modeling. *Nat Biotechnol* 38: 1408–1414

Blank V (2008) Small Maf proteins in mammalian gene control: mere dimerization partners or dynamic transcriptional regulators? *J Mol Biol* 376: 913–925

Blériot C, Chakarov S, Ginhoux F (2020) Determinants of resident tissue macrophage identity and function. *Immunity* 52: 957–970

Bonnardel J, Da Silva C, Henri S, Tamoutounour S, Chasson L, Montañana-Sanchis F, Gorvel J-P, Lelouard H (2015) Innate and adaptive immune functions of Peyer's patch monocyte-derived cells. *Cell Rep* 11: 770–784

Briseño CG, Haldar M, Kretzer NM, Wu X, Theisen DJ, Kc W, Durai V, Grajales-Reyes GE, Iwata A, Bagadia P *et al* (2016) Distinct transcriptional programs control cross-priming in classical and monocyte-derived dendritic cells. *Cell Rep* 15: 2462–2474

Calderon B, Carrero JA, Ferris ST, Sojka DK, Moore L, Epelman S, Murphy KM, Yokoyama WM, Randolph GJ, Unanue ER (2015) The pancreas anatomy conditions the origin and properties of resident macrophages. *J Exp Med* 212: 1497–1512

Chopin M, Lun AT, Zhan Y, Schreuder J, Coughlan H, D'Amico A, Mielke LA, Almeida FF, Kueh AJ, Dickins RA *et al* (2019) Transcription factor PU.1 promotes conventional dendritic cell identity and function via induction of transcriptional regulator DC-SCRIPT. *Immunity* 50: 77–90.e5

Coillard A, Segura E (2019) *In vivo* differentiation of human monocytes. *Front Immunol* 10: 1–7

Coillard A, Guyonnet L, De Juan A, Cros A, Segura E (2021) TLR or NOD receptor signaling skews monocyte fate decision via distinct mechanisms driven by mTOR and miR-155. *Proc Natl Acad Sci USA* 118: e2109225118

Colamonici OR, Domanski P, Sweitzer SM, Larner A, Buller RML (1995) Vaccinia virus B18R gene encodes a type I interferon-binding protein that blocks interferon α transmembrane signaling. *J Biol Chem* 270: 15974–15978

Devalaraja S, To TKJ, Folkert IW, Natesan R, Alam MZ, Li M, Tada Y, Budagyan K, Dang MT, Zhai L *et al* (2020) Tumor-derived retinoic acid regulates intratumoral monocyte differentiation to promote immune suppression. *Cell* 180: 1098–1114.e16

Epelman S, Lavine KJ, Beaudin AE, Sojka DK, Carrero JA, Calderon B, Brija T, Gautier EL, Ivanov S, Satpathy AT *et al* (2014) Embryonic and adult-derived resident cardiac macrophages are maintained through distinct mechanisms at steady state and during inflammation. *Immunity* 40: 91–104

Garcia-Alonso L, Holland CH, Ibrahim MM, Turei D, Saez-Rodriguez J (2019) Benchmark and integration of resources for the estimation of human transcription factor activities. *Genome Res* 29: 1363–1375

Goudot C, Coillard A, Villani AC, Gueguen P, Cros A, Sarkizova S, Tang-Huau TL, Bohec M, Baulande S, Hacohen N *et al* (2017) Aryl hydrocarbon receptor controls monocyte differentiation into dendritic cells versus macrophages. *Immunity* 47: 582–596.e6

Guilliams M, Mildner A, Yona S (2018) Developmental and functional heterogeneity of monocytes. *Immunity* 49: 595–613

Guilliams M, Thierry GR, Bonnardel J, Bajenoff M (2020) Establishment and maintenance of the macrophage niche. *Immunity* 52: 434–451

Hao Y, Hao S, Andersen-Nissen E, Mauck WM, Zheng S, Butler A, Lee MJ, Wilk AJ, Darby C, Zager M *et al* (2021) Integrated analysis of multimodal single-cell data. *Cell* 184: 3573–3587.e29

Holland CH, Tanevski J, Perales-Patón J, Gleixner J, Kumar MP, Mereu E, Joughin BA, Stegle O, Lauffenburger DA, Heyn H *et al* (2020) Robustness and applicability of transcription factor and pathway analysis tools on single-cell RNA-seq data. *Genome Biol* 21: 36

Jakubzick CV, Randolph GJ, Henson PM (2017) Monocyte differentiation and antigen-presenting functions. *Nat Rev Immunol* 17: 349–362

Kamijo R, Harada H, Matsuyama T, Bosland M, Gerecitano J, Shapiro D, Le J, Koh SI, Kimura T, Green SJ *et al* (1994) Requirement for transcription factor IRF-1 in NO synthase induction in macrophages. *Science* 263: 1612–1615

Katsuoka F, Yamamoto M (2016) Small Maf proteins (MafF, MafG, MafK): history, structure and function. *Gene* 586: 197–205

Kim K-W, Williams JW, Wang Y-T, Ivanov S, Gilfillan S, Colonna M, Virgin HW, Gautier EL, Randolph GJ (2016) MHC II+ resident peritoneal and pleural macrophages rely on IRF4 for development from circulating monocytes. *J Exp Med* 213: 1951–1959

La Manno G, Soldatov R, Zeisel A, Braun E, Hochgerner H, Petukhov V, Lidschreiber K, Kastriti ME, Lönnerberg P, Furlan A *et al* (2018) RNA velocity of single cells. *Nature* 560: 494–498

Langlais D, Barreiro LB, Gros P (2016) The macrophage IRF8/IRF1 regulome is required for protection against infections and is associated with chronic inflammation. *J Exp Med* 213: 585–603

Levine JH, Simonds EF, Bendall SC, Davis KL, Amir ED, Tadmor MD, Litvin O, Fienberg HG, Jager A, Zunder ER *et al* (2015) Data-driven phenotypic dissection of AML reveals progenitor-like cells that correlate with prognosis. *Cell* 162: 184–197

Liu Z, Gu Y, Chakarov S, Bleriot C, Kwok I, Chen X, Shin A, Huang W, Dress RJ, Dutertre C-A *et al* (2019) Fate mapping via Ms4a3-expression history traces monocyte-derived cells. *Cell* 178: 1509–1525.e19

Mendes K, Schmidhofer S, Minderjahn J, Glatz D, Kiesewetter C, Raithel J, Wimmer J, Gebhard C, Rehli M (2021) The epigenetic pioneer EGR2 initiates DNA demethylation in differentiating monocytes at both stable and transient binding sites. *Nat Commun* 12: 1556

Menezes S, Melandri D, Anselmi G, Perchet T, Loschko J, Dubrot J, Patel R, Gautier EL, Hugues S, Longhi MP *et al* (2016) The heterogeneity of Ly6Chi monocytes controls their differentiation into iNOS+ macrophages or monocyte-derived dendritic cells. *Immunity* 45: 1205–1218

Mildner A, Schönheit J, Giladi A, David E, Lara-Astiaso D, Lorenzo-Vivas E, Paul F, Chappell-Maor L, Priller J, Leutz A *et al* (2017) Genomic characterization of murine monocytes reveals C/EBPβ transcription factor dependence of Ly6C− cells. *Immunity* 46: 849–862.e7

Molawi K, Wolf Y, Kandalla PK, Favret J, Hagemeyer N, Frenzel K, Pinto AR, Klapproth K, Henri S, Malissen B *et al* (2014) Progressive replacement of embryo-derived cardiac macrophages with age. *J Exp Med* 211: 2151–2158

Place DE, Malireddi RKS, Kim J, Vogel P, Yamamoto M, Kanneganti T-D (2021) Osteoclast fusion and bone loss are restricted by interferon inducible guanylate binding proteins. *Nat Commun* 12: 496

Rosain J, Neehus AL, Manry J, Yang R, Le Pen J, Daher W, Liu Z, Chan YH, Tahuil N, Türel Ö *et al* (2023) Human IRF1 governs macrophagic IFN-γ immunity to mycobacteria. *Cell* 186: 621–645.e33

Salem S, Gao C, Li A, Wang H, Nguyen-Yamamoto L, Goltzman D, Henderson JE, Gros P (2014) A novel role for interferon regulatory factor 1 (IRF1) in regulation of bone metabolism. *J Cell Mol Med* 18: 1588–1598

Salix B, Jean-François D, Livia O, Adam-Nicolas P, Jin YT, Sylvie L, Cheolho C, Nathalie L (2019) The orphan nuclear receptor NR4A3 controls the differentiation of monocyte-derived dendritic cells following microbial stimulation. *Proc Natl Acad Sci USA* 116: 15150–15159

Sander J, Schmidt SV, Cirovic B, McGovern N, Papantonopoulou O, Hardt AL, Aschenbrenner AC, Kreer C, Quast T, Xu AM *et al* (2017) Cellular differentiation of human monocytes is regulated by time-dependent Interleukin-4 signaling and the transcriptional regulator NCOR2. *Immunity* 47: 1051–1066.e12

Shaw TN, Houston SA, Wemyss K, Bridgeman HM, Barbera TA, Zangerle-Murray T, Strangward P, Ridley AJL, Wang P, Tamoutounour S *et al* (2018) Tissue-resident macrophages in the intestine are long lived and defined by Tim-4 and CD4 expression. *J Exp Med* 215: 1507–1518

Shengbo Z, Coughlan HD, Ashayeripanah M, Seizova S, Kueh AJ, Brown DV, Cao W, Jacquelot N, D'Amico A, Lew AM *et al* (2021) Type 1 conventional dendritic cell fate and function are controlled by DC-SCRIPT. *Sci Immunol* 6: eabf4432

Song R, Gao Y, Dozmorov I, Malladi V, Saha I, McDaniel MM, Parameswaran S, Liang C, Arana C, Zhang B *et al* (2021) IRF1 governs the differential interferon-stimulated gene responses in human monocytes and macrophages by regulating chromatin accessibility. *Cell Rep* 34: 108891

Street K, Risso D, Fletcher RB, Das D, Ngai J, Yosef N, Purdom E, Dudoit S (2018) Slingshot: cell lineage and pseudotime inference for single-cell transcriptomics. *BMC Genomics* 19: 477

Tamoutounour S, Guilliams M, MontananaSanchis F, Liu H, Terhorst D, Malosse C, Pollet E, Ardouin L, Luche H, Sanchez C *et al* (2013) Origins and functional specialization of macrophages and of conventional and monocyte-derived dendritic cells in mouse skin. *Immunity* 39: 925–938

Tang-Huau TL, Gueguen P, Goudot C, Durand M, Bohec M, Baulande S, Pasquier B, Amigorena S, Segura E (2018) Human *in vivo*-generated monocyte-derived dendritic cells and macrophages cross-present antigens through a vacuolar pathway. *Nat Commun* 9: 2570

Testa U, Stellacci E, Pelosi E, Sestili P, Venditti M, Orsatti R, Fragale A, Petrucci E, Pasquini L, Belardelli F *et al* (2004) Impaired myelopoiesis in mice devoid of interferon regulatory factor 1. *Leukemia* 18: 1864–1871

Triantis V, Trancikova DE, Looman MWG, Hartgers FC, Janssen RAJ, Adema GJ (2006) Identification and characterization of DC-SCRIPT, a novel dendritic

 © 2023 The Authors

cell-expressed member of the zinc finger family of transcriptional regulators. *J Immunol* 176: 1081–1089

Vento-Tormo R, Company C, Rodríguez-Ubreva J, de la Rica L, Urquiza JM, Javierre BM, Sabarinathan R, Luque A, Esteller M, Aran JM *et al* (2016) IL-4 orchestrates STAT6-mediated DNA demethylation leading to dendritic cell differentiation. *Genome Biol* 17: 4

Villani A-C, Satija R, Reynolds G, Sarkizova S, Shekhar K, Fletcher J, Griesbeck M, Butler A, Zheng S, Lazo S *et al* (2017) Single-cell RNA-seq reveals new types of human blood dendritic cells, monocytes, and progenitors. *Science* 356: eaah4573

Villar J, Cros A, De Juan A, Alaoui L, Bonte PE, Lau CM, Tiniakou I, Reizis B, Segura E (2022) ETV3 and ETV6 enable monocyte differentiation into dendritic cells by repressing macrophage fate commitment. *Nat Immunol* 24: 84–95

Zigmond E, Varol C, Farache J, Elmaliah E, Satpathy AT, Friedlander G, Mack M, Shpigel N, Boneca IG, Murphy KM *et al* (2012) Ly6Chi monocytes in the inflamed colon give rise to proinflammatory effector cells and migratory antigen-presenting cells. *Immunity* 37: 1076–1090

License: This is an open access article under the terms of the Creative Commons Attribution License, which permits use, distribution and reproduction in any medium, provided the original work is properly cited.

Expanded View Figures

Figure EV1. Features of the scRNA-seq dataset.

A, B UMAP of integrated scRNAseq data from all time points. The donor origin of cells is shown. (B) Percentage of cells from each donor at different time points. Total number of cells is shown at the top of each bar.
C Number of genes (Features), counts (*n*Counts) and percentage of mitochondrial genes in scRNA-seq data for all time points and split by donor.
D Unsupervised clustering of all integrated data at resolution 0.2.
E Identification of possible contaminants. Enrichment of B cell, NK and T cell signatures is shown by clusters. Contaminating cells (2.8% of all cells) were excluded from subsequent analysis.
F–I Remapping of the data without contaminating cells. (F) UMAP of integrated scRNAseq data from all time points. The donor origin of cells is shown. (G) The time point origin of cells is shown. (H) Top differentially expressed genes between timepoints. (I) UMAP colored by RNA velocity pseudotime obtained with scVelo.

 © 2023 The Authors

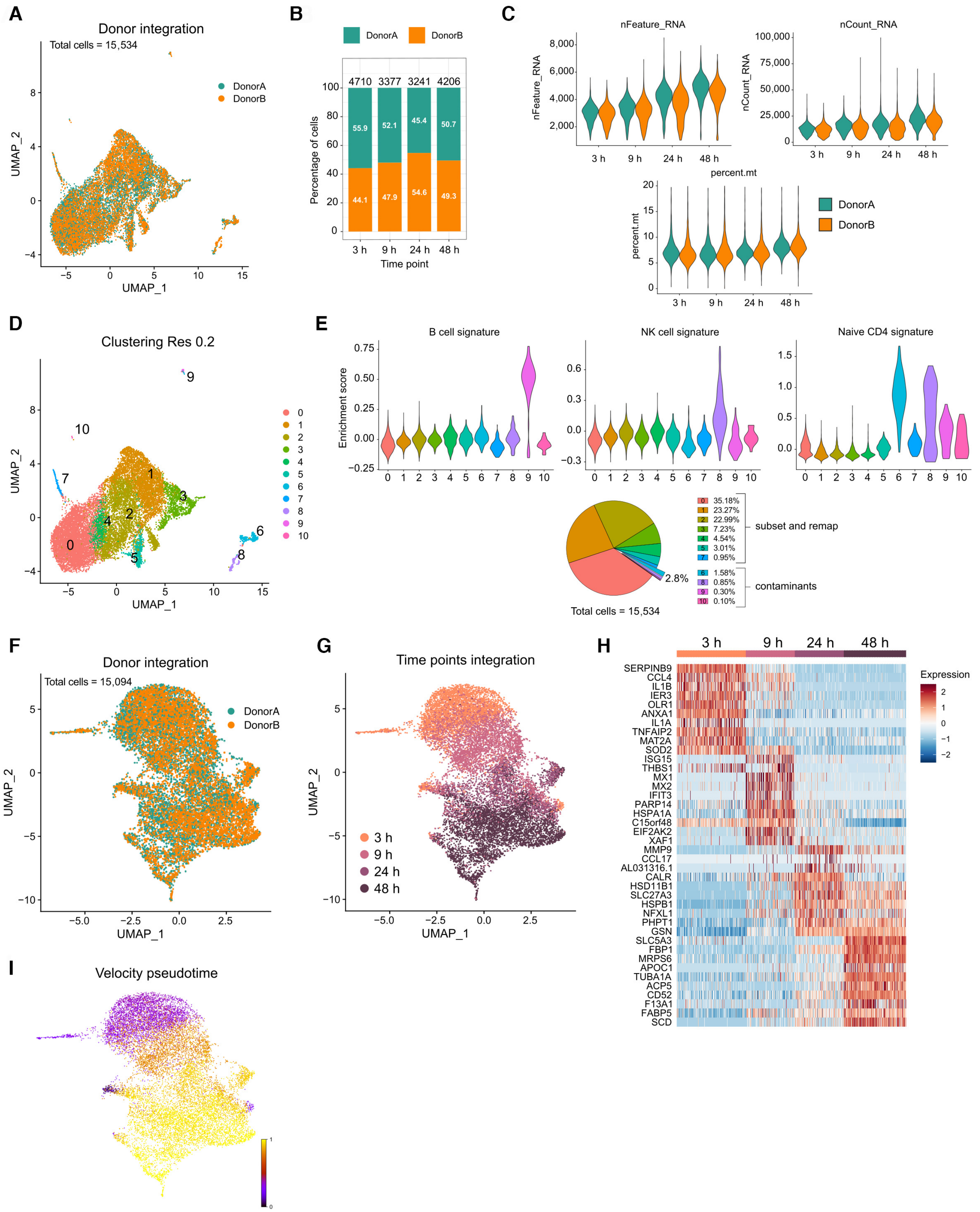
A
Donor integration
Total cells = 15,534
DonorA
DonorB
UMAP_1
UMAP_2
B
DonorA
DonorB
4710 3377 3241 4206
55.9 52.1 45.4 50.7
44.1 47.9 54.6 49.3
Percentage of cells
Time point
C
nFeature_RNA
nCount_RNA
percent.mt
DonorA
DonorB
D
Clustering Res 0.2
E
B cell signature
NK cell signature
Naive CD4 signature
Enrichment score
35.18%
23.27%
22.99%
7.23%
4.54%
3.01%
0.95%
subset and remap
1.58%
0.85%
0.30%
0.10%
contaminants
2.8%
Total cells = 15,534
F
Donor integration
Total cells = 15,094
G
Time points integration
3 h
9 h
24 h
48 h
H
Expression
SERPINB9
CCL4
IL1B
IER3
OLR1
ANXA1
IL1A
TNFAIP2
MAT2A
SOD2
ISG15
THBS1
MX1
MX2
IFIT3
PARP14
HSPA1A
C15orf48
EIF2AK2
XAF1
MMP9
CCL17
AL031316.1
CALR
HSD11B1
SLC27A3
HSPB1
NFXL1
PHPT1
GSN
SLC5A3
FBP1
MRPS6
APOC1
TUBA1A
ACP5
CD52
F13A1
FABP5
SCD
I
Velocity pseudotime

Figure EV1.

Figure EV2. Features of the peritonitis model.

A–C Peritonitis was induced by intra-peritoneal injection of thioglycolate. (A) $CD45.2^+$ $Ly6C^+$ monocytes were transferred into the inflamed peritoneum of $CD45.1^+$ mice. Strategy for the analysis of the time-course dataset. (B–C) Cells from the peritoneal lavage were analyzed by flow cytometry after 2 days. (B) Gating strategy is shown. (C) Expression of indicated markers. Representative results from one mouse are shown (n = 6 biological replicates). FMO = fluorescence-minus-one control.

D Purity of the bone marrow monocytes isolated with a magnetic kit. Representative results from one mouse are shown (n = 5 biological replicates). Percentage of monocytes, neutrophils and DC precursors in the purified cells (mean ± SD is shown).

© 2023 The Authors

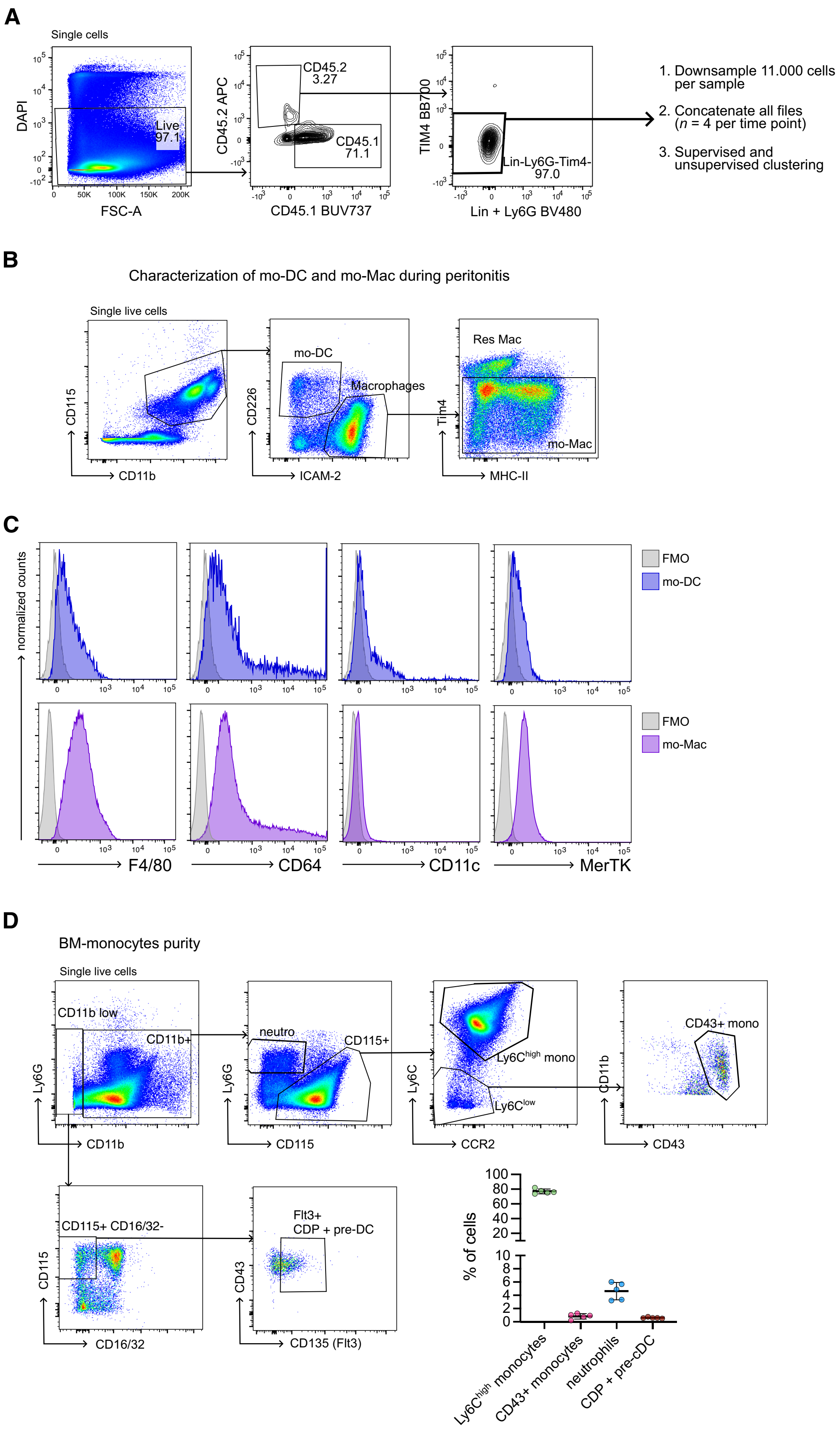
A
Single cells
DAPI
FSC-A
Live 97.1
CD45.2 APC
CD45.1 BUV737
CD45.2 3.27
CD45.1 71.1
TIM4 BB700
Lin + Ly6G BV480
Lin-Ly6G-Tim4- 97.0
1. Downsample 11.000 cells per sample
2. Concatenate all files (n = 4 per time point)
3. Supervised and unsupervised clustering
B
Characterization of mo-DC and mo-Mac during peritonitis
Single live cells
CD115
CD11b
CD226
ICAM-2
mo-DC
Macrophages
Tim4
MHC-II
Res Mac
mo-Mac
C
normalized counts
FMO
mo-DC
FMO
mo-Mac
F4/80
CD64
CD11c
MerTK
D
BM-monocytes purity
Single live cells
CD11b low
CD11b+
Ly6G
CD11b
neutro
CD115+
CD115
Ly6C
CCR2
Ly6Chigh mono
Ly6Clow
CD11b
CD43
CD43+ mono
CD115+ CD16/32-
CD115
CD16/32
Flt3+
CDP + pre-DC
CD43
CD135 (Flt3)
% of cells
Ly6Chigh monocytes
CD43+ monocytes
neutrophils
CDP + pre-cDC

Figure EV2.
