# Supplementary material for: Monocytes differentiate along two alternative pathways during sterile inflammation
Source: EMBO Rep. 2023 May 16;24(7):e56308. doi: 10.15252/embr.202256308 (PMC10328069; doi:10.15252/embr.202256308)
Supplement: Supplementary file 6 — Source Data for Figure 4 [file EMBR-24-e56308-s006.zip › figure 4/4B/source data 4B.pdf]

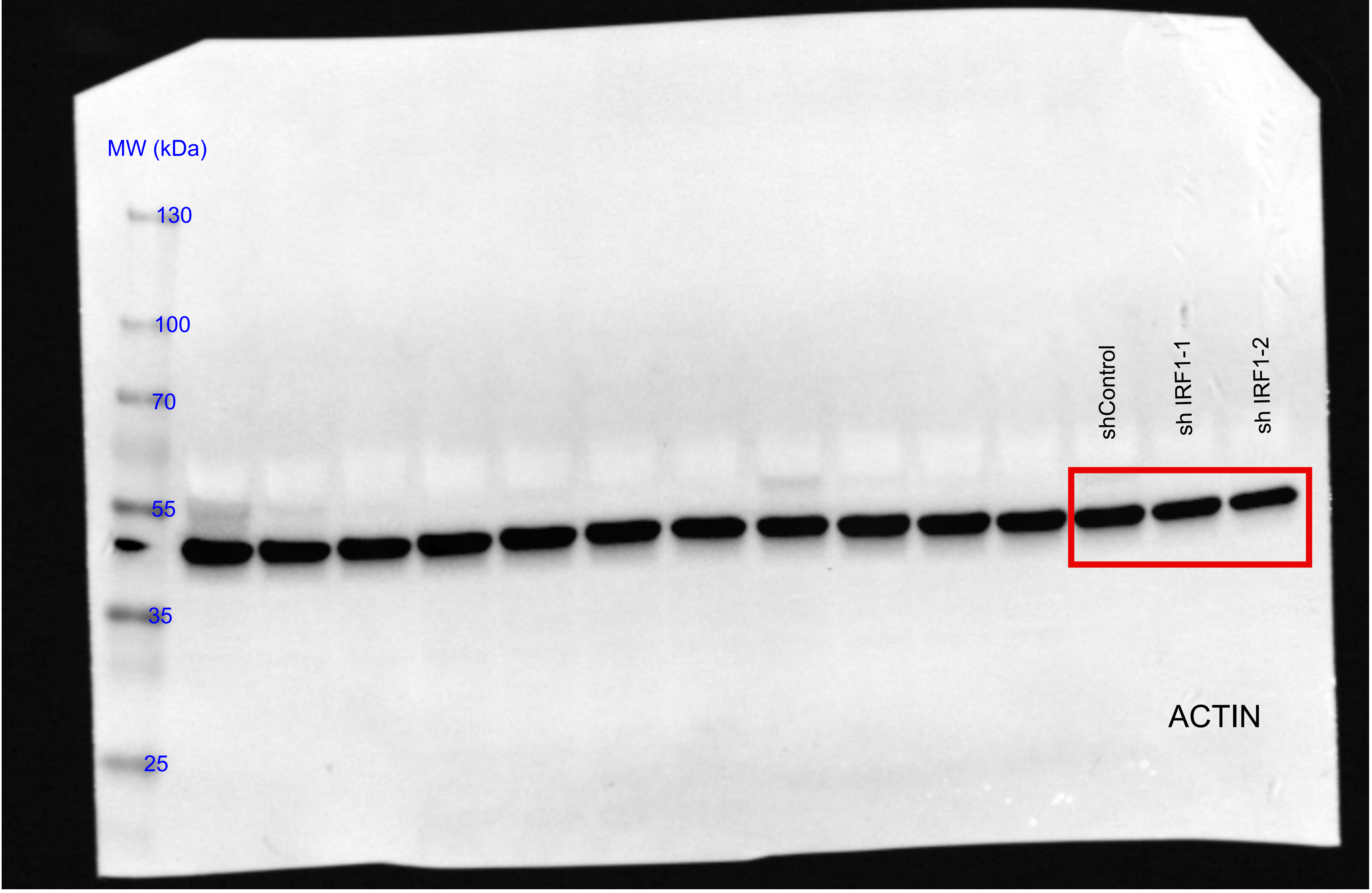
MW (kDa)
130
100
70
55
35
25
shControl
sh IRF1-1
sh IRF1-2
ACTIN

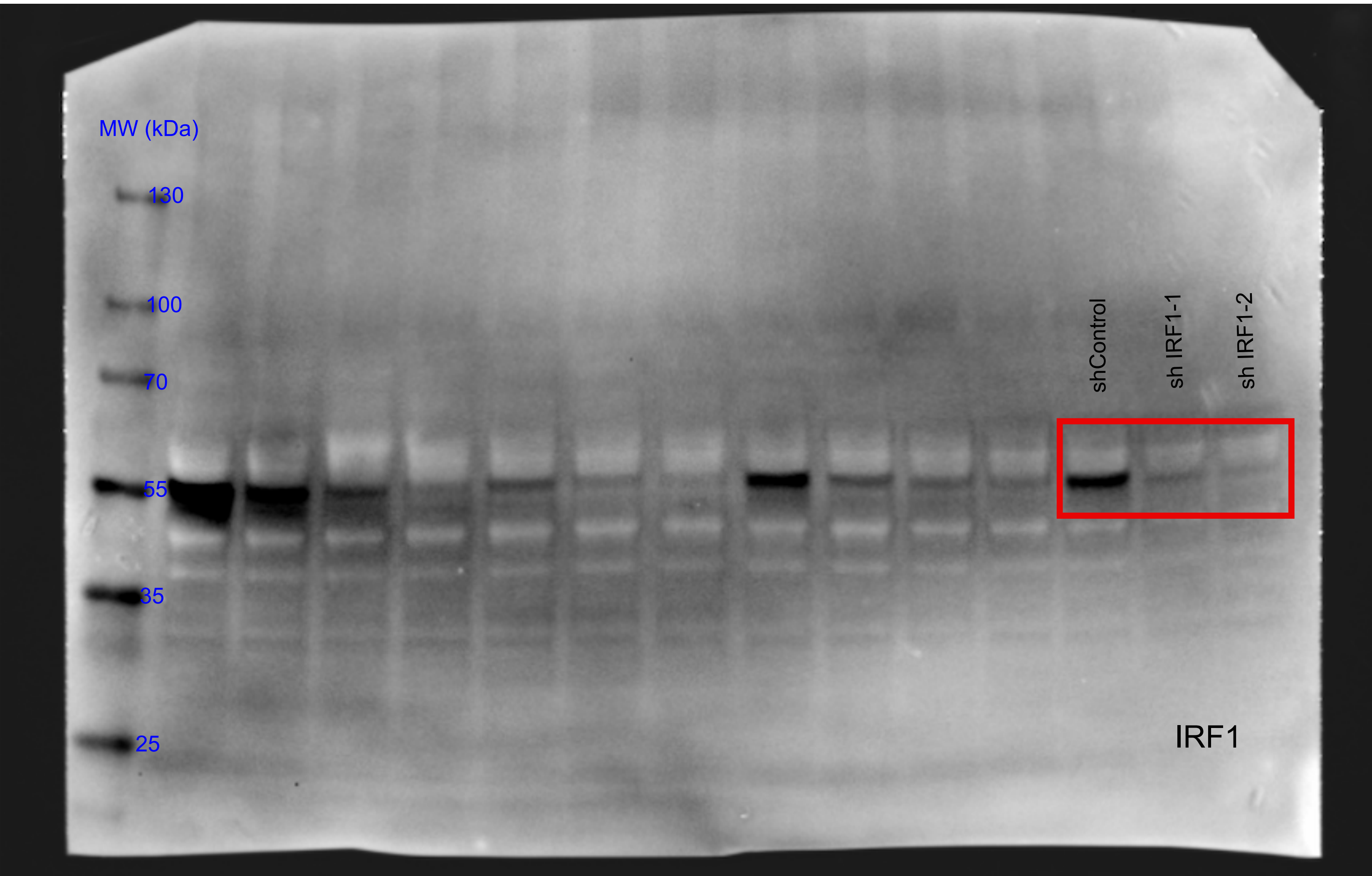
MW (kDa)
130
100
70
55
35
25
shControl
sh IRF1-1
sh IRF1-2
IRF1
