# Supplementary figures and images for: Monocytes differentiate along two alternative pathways during sterile inflammation
Source: EMBO Rep. 2023 May 16;24(7):e56308. doi: 10.15252/embr.202256308 (PMC10328069; doi:10.15252/embr.202256308)

day 0 day 1 day 2 day 5

MW (kDa)

70

55

35

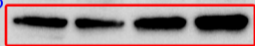

Actin

day 0 day 1 day 2 day 5

MW (kDa)

70

55

35

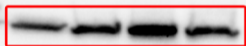

IRF1

Supplement: Supplementary file 6 — Source Data for Figure 4 [file EMBR-24-e56308-s006.zip › figure 4/4A/source data 4A.pdf]

day 0 day 1 day 2 day 5

MW (kDa)

70

55

35

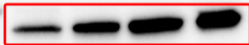

Actin

day 0 day 1 day 2 day 5

MW (kDa)

70

55

35

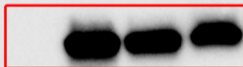

MAFF

Supplement: Supplementary file 7 — Source Data for Figure 5 [file EMBR-24-e56308-s005.zip › figure 5/5G/source data 5G.pdf]

sh Control  
sh MAFF-1  
sh MAFF-2

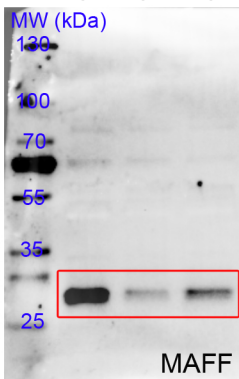

sh Control  
sh MAFF-1  
sh MAFF-2

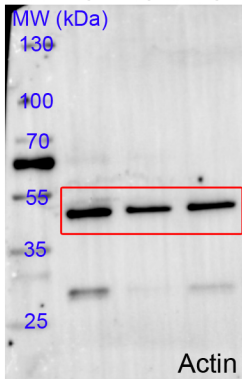

Supplement: Supplementary file 7 — Source Data for Figure 5 [file EMBR-24-e56308-s005.zip › figure 5/5H/source data 5H.pdf]

day 0 day 1 day 2 day 5

MW (kDa)

70

55

35

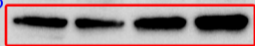

Actin

day 0 day 1 day 2 day 5

MW (kDa)

70

55

35

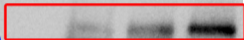

ZNF366

Supplement: Supplementary file 7 — Source Data for Figure 5 [file EMBR-24-e56308-s005.zip › figure 5/5C/source data 5C.pdf]

sh Control  
sh ZNF366-1  
sh ZNF366-2

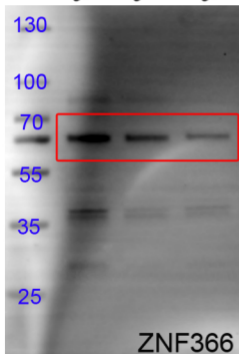

MW (kDa)

sh Control  
sh ZNF366-1  
sh ZNF366-2

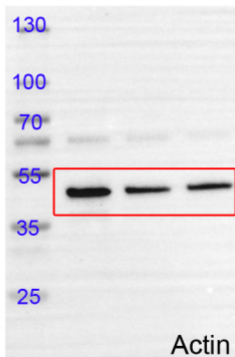

MW (kDa)

Supplement: Supplementary file 7 — Source Data for Figure 5 [file EMBR-24-e56308-s005.zip › figure 5/5D/source data 5D.pdf]
