# Supplementary material for: Monocytes differentiate along two alternative pathways during sterile inflammation
Source: EMBO Rep. 2023 May 16;24(7):e56308. doi: 10.15252/embr.202256308 (PMC10328069; doi:10.15252/embr.202256308)
Supplement: Supplementary file 8 [file EMBR-24-e56308-s004.pdf]

EMBO reports

Monocytes differentiate along two alternative pathways during sterile inflammation

Javiera Villar, Léa Ouaknin, Adeline Cros, and Elodie Segura
DOI: 10.15252/embr.202256308

Corresponding author(s): Elodie Segura (elodie.segura@curie.fr)

Review Timeline:

Submission Date:	16th Oct 22
Editorial Decision:	9th Nov 22
Revision Received:	27th Mar 23
Editorial Decision:	11th Apr 23
Revision Received:	18th Apr 23
Accepted:	2nd May 23

Editor: Achim Breiling

Transaction Report:

(Note: With the exception of the correction of typographical or spelling errors that could be a source of ambiguity, letters and reports are not edited. Depending on transfer agreements, referee reports obtained elsewhere may or may not be included in this compilation. Referee reports are anonymous unless the Referee chooses to sign their reports.)

Dear Dr. Segura,

Thank you for the submission of your research manuscript to EMBO reports. I have now received the reports from the three referees that were asked to evaluate your study, which can be found at the end of this email.

As you will see, referees #2 and #3 think that these findings are of interest. However, they have several comments, concerns, and suggestions, indicating that a major revision of the manuscript is necessary to allow publication of the study in EMBO reports. As the reports are below, and all the referee concerns need to be addressed, I will not detail them here.

Given the constructive referee comments, we would like to invite you to revise your manuscript with the understanding that all referee concerns must be addressed in the revised manuscript and/or in a detailed point-by-point response. Acceptance of your manuscript will depend on a positive outcome of a second round of review. It is EMBO reports policy to allow a single round of revision only and acceptance of the manuscript will therefore depend on the completeness of your responses included in the next, final version of the manuscript.

Revised manuscripts should be submitted within three months of a request for revision. Please contact me to discuss the revision (also by video chat) if you have questions or comments regarding the revision, or should you need additional time.

When submitting your revised manuscript, please also carefully review the instructions that follow below.

PLEASE NOTE THAT upon resubmission revised manuscripts are subjected to an initial quality control prior to exposition to re-review. Upon failure in the initial quality control, the manuscripts are sent back to the authors, which may lead to delays. Frequent reasons for such a failure are the lack of the data availability section (please see below) and the presence of statistics based on n=2 (the authors are then asked to present scatter plots or provide more data points).

When submitting your revised manuscript, we will require:

1) a .docx formatted version of the final manuscript text (including legends for main figures, EV figures and tables), but without the figures included. Figure legends should be compiled at the end of the manuscript text.

2) individual production quality figure files as .eps, .tif, .jpg (one file per figure), of main figures (up to 8) and EV figures. Please upload these as separate, individual files upon re-submission.

The Expanded View format, which will be displayed in the main HTML of the paper in a collapsible format, has replaced the Supplementary information. You can submit up to 5 images as Expanded View. Please follow the nomenclature Figure EV1, Figure EV2 etc. The figure legend for these should be included in the main manuscript document file in a section called Expanded View Figure Legends after the main Figure Legends section. Additional Supplementary material should be supplied as a single pdf file labeled Appendix. The Appendix should have page numbers and needs to include a table of content on the first page (with page numbers) and legends for all content. Please follow the nomenclature Appendix Figure Sx, Appendix Table Sx etc. throughout the text, and also label the figures and tables according to this nomenclature.

For more details, please refer to our guide to authors:
http://www.embopress.org/page/journal/14693178/authorguide#manuscriptpreparation

Please consult our guide for figure preparation:
http://wol-prod-cdn.literatumonline.com/pb-assets/embo-site/EMBOPress_Figure_Guidelines_061115-1561436025777.pdf

See also the guidelines for figure legend preparation:
https://www.embopress.org/page/journal/14693178/authorguide#figureformat

3) a complete author checklist, which you can download from our author guidelines (https://www.embopress.org/page/journal/14693178/authorguide). Please insert page numbers in the checklist to indicate where the requested information can be found in the manuscript. The completed author checklist will also be part of the RPF.

Please also follow our guidelines for the use of living organisms, and the respective reporting guidelines:
http://www.embopress.org/page/journal/14693178/authorguide#livingorganisms

4) that primary datasets produced in this study (e.g. RNA-seq, ChIP-seq, structural and array data) are deposited in an appropriate public database. If no primary datasets have been deposited, please also state this in a dedicated section (e.g. 'No primary datasets have been generated and deposited'), see below.

See also: http://embor.embopress.org/authorguide#datadeposition

Please remember to provide a reviewer password if the datasets are not yet public.

The accession numbers and database should be listed in a formal "Data Availability" section (placed after Materials & Methods) that follows the model below. This is now mandatory (like the COI statement). Please note that the Data Availability Section is restricted to new primary data that are part of this study. This section is mandatory. As indicated above, if no primary datasets have been deposited, please state this in this section

Data availability

The datasets produced in this study are available in the following databases:

- RNA-Seq data: Gene Expression Omnibus GSE46843 (https://www.ncbi.nlm.nih.gov/geo/query/acc.cgi?acc=GSE46843)
- [data type]: [name of the resource] [accession number/identifier/doi] ([URL or identifiers.org/DATABASE:ACCESSION])

*** Note - All links should resolve to a page where the data can be accessed. ***

Moreover, I have these editorial requests:

6) We now request the publication of original source data with the aim of making primary data more accessible and transparent to the reader. Our source data coordinator will contact you to discuss which figure panels we would need source data for and will also provide you with helpful tips on how to upload and organize the files.

7) Our journal encourages inclusion of *data citations in the reference list* to directly cite datasets that were re-used and obtained from public databases. Data citations in the article text are distinct from normal bibliographical citations and should directly link to the database records from which the data can be accessed. In the main text, data citations are formatted as follows: "Data ref: Smith et al, 2001" or "Data ref: NCBI Sequence Read Archive PRJNA342805, 2017". In the Reference list, data citations must be labeled with "[DATASET]". A data reference must provide the database name, accession number/identifiers and a resolvable link to the landing page from which the data can be accessed at the end of the reference. Further instructions are available at: http://www.embopress.org/page/journal/14693178/authorguide#referencesformat

8) Regarding data quantification and statistics, please make sure that the number "n" for how many independent experiments were performed, their nature (biological versus technical replicates), the bars and error bars (e.g. SEM, SD) and the test used to calculate p-values is indicated in the respective figure legends (also for potential EV figures and all those in the final Appendix). Please also check that all the p-values are explained in the legend, and that these fit to those shown in the figure. Please provide statistical testing where applicable. Please avoid the phrase 'independent experiment', but clearly state if these were biological or technical replicates. Please also indicate (e.g. with n.s.) if testing was performed, but the differences are not significant. In case n=2, please show the data as separate datapoints without error bars and statistics. See also: http://www.embopress.org/page/journal/14693178/authorguide#statisticalanalysis

9) Please also note our reference format:
http://www.embopress.org/page/journal/14693178/authorguide#referencesformat

10) We updated our journal's competing interests policy in January 2022 and request authors to consider both actual and perceived competing interests. Please review the policy https://www.embopress.org/competing-interests and update your competing interests if necessary. Please name this section 'Disclosure and Competing Interests Statement' and put it after the Acknowledgements section.

11) We now use CRediT to specify the contributions of each author in the journal submission system. CRediT replaces the author contribution section. Please use the free text box to provide more detailed descriptions. See also guide to authors: https://www.embopress.org/page/journal/14693178/authorguide#authorshipguidelines

12) Please order the manuscript sections like this, using these names:
Title page - Abstract - Keywords - Introduction - Results - Discussion - Materials and Methods - Data availability section - Acknowledgements (including funding information) - Disclosure and Competing Interests Statement - References - Figure legends - Expanded View Figure legends

I look forward to seeing a revised version of your manuscript when it is ready. Please let me know if you have questions or comments regarding the revision.

Please use this link to submit your revision: https://embor.msubmit.net/cgi-bin/main.plex

Yours sincerely,

Achim Breiling
Senior Editor
EMBO Reports

Referee #1:

This study by Villar et al. examined mo-Mac versus mo-DC differentiation pathway in both human and mouse models. The authors rely on temporal single-cell RNA sequencing followed by RNA velocity analysis using scVelo (human, in vitro) and pseudotime trajectory analysis using Slingshot (mouse, in vivo) to get the conclusion that, mo-Mac and mo-DC develop via two divergent and alternative differentiation pathways. In addition to the author's previous discoveries, this study further identified the transcription factors IRF1, ZNF366 and MAFF to be regulators of mo-Mac and mo-DC differentiation.

While RNA velocity analysis and pseudotime trajectory analysis can suggest potential developmental pathway, these computational methods alone cannot serve as the definitive evidence to prove the "divergent and alternative differentiation pathways". The transcription factors IRF1, ZNF366 and MAFF were only partially required for mo-Mac or mo-DC differentiation, and the effect of IRF1 is tested in a culture condition that only generate mo-Mac but not mo-DC.

Overall, this study does not have strong enough evidence to support the conclusion that "mo-Mac and mo-DC represent two alternative cell fates requiring distinct transcription factors for their differentiation".

Referee #2:

The paper by Villar and colleagues tackles the long-lasting question about the differentiation of monocytes into macrophages and dendritic cells. The authors use an in vitro approach to investigate the temporal differentiation of monocytes by scRNA-Seq. Based on RNA velocity analysis, the authors claim to identify a splitting point, in which monocytes either develop into DCs or macrophages. Additionally they profile monocyte-derived cells at different time-points after transfer into the peritoneum of thioglycolate-injected animals by flow cytometry and found a similar bifurcation. The authors extracted three transcription factors that might play a role in this separation, namely Irf1, Znf366 and MAFF. Indeed, silencing each one of them let to reduced macrophage and DC development, respectively.

The experiments are well-performed and the writing is clear. However, there are some important things that need be addressed:

1. For the experiments represented in Fig. 2 the authors isolated monocytes from the BM with an isolation kit. The company of this kit gives no information about the antibodies used to select lineage positive cells nor do the authors show the purity of cells after isolation. Since the three hours UMAP blot in Fig. 2C suggest that maybe 50% of CD45.2+ cells are Ly6C- (corresponding to clusters 2/3/14 in 2G), it is important to know, if this reflects down-regulation or contamination with other BM cells such as precursors or DCs. This is of importance cause these cells could represent an alternative precursor subset for moDC or macrophages. What happens for instance, when the authors use cluster 2 instead of cluster 12 as starting point for their slingshot analysis? FACS-sorted cells should be used for these kind of experiments.

2. It is unclear, how the authors identified IRF1, ZNF366 and MAFF as possible candidates in Fig. 3. What is the logic of the selection? The authors mention: 'we selected for further study IRF1, which is more expressed in macrophage-engaged clusters 4 and 6 and shows high transcriptional activity in the clusters 5 and 6'. Isn't the same true for STAT2, IRF9 and STAT1? Why didn't they choose these genes? MAFF and ZNF366 are also not listed in Fig. 3E and their selection is even not explained. Maybe an analysis using LISA (http://lisa.cistrome.org/) might help to justify their choice.

3. The authors show in Fig. 4B that IRF1 influences macrophage development by reducing the frequency of CD16+ cells two-fold. Later experiments in this figure suggest that the expression of ISG genes does not influence moDC development. Accordingly, the authors should additionally show, how IRF1 silencing influences the differentiation of cells under M4T conditions. A cell quantification rather than a frequency analysis should be performed to show that moDC development is unaffected by IRF1 silencing in M4T-treated cells. The authors should also add IRF1 expression to the real time PCR quantification shown in Fig. 4E to examine the expression changes based on culture condition.

4. The mechanism, how IRF1, ZNF366 and MAFF influence the development of monocytes is completely unclear. Maybe a molecular analysis is too much to ask for, even though feasible, but at least the survival and apoptosis rate of monocyte-derived cells after gene silencing at different time points should be analysed.

Minor things:

-The current title emphasises that the here identified transcriptional program is conserved in all monocyte to macrophage or DC transition scenarios. This, however, seems to be a generalisation. As mentioned by the authors themselves, there finding might

not be true during neuroinflammation. Accordingly, the title should be adjusted to fit the actual findings (which is under in vitro conditions and during peritonitis)

-In respect to Fig. 1 the authors write on page 5: 'these results reveal two divergent and alternative differentiation pathways'. This statement is solely based on RNA velocity analysis. However, RNA velocity is an algorithm that relies on the assumption that a molecular connection between cells really exists. Time point analysis of RBC differentiation showed that the RNA velocity alone is not able to predict an experimental induced differentiation path, at least not with adjustments (Barile et al., 2021). Therefore RNA velocity cannot replace experimental proof and is just a possible interpretation of biological findings. Accordingly it should not be stated that 'these results reveal (...) differentiation' but instead 'these results suggest (...) differentiation'.

-Could the authors add IRF1, MAFF and ZNF366 to the UMAP represented in Fig. 1D or to later figures? A time-dependent validation of gene expression with real-time PCR should be added as well.

-Also the expression of other small MAF family members should be represented as UMAPs.

-The authors write in figure legend 5D that the "Median is shown (n=8 in 3 independent experiments)", but the blot shows five data points. The data points mentioned in figure legend 5G also do not fit to the data shown in the figure. Can the authors clarify this point.

-It is not completely clear, if 5-days cultured monocytes were used for Immunoblot analysis shown in Fig. 5C and 5F. Are ZNF366 and MAFF expressed under steady state conditions? An Immunoblot analysis of freshly isolated monocytes should be added.

Referee #3:

Monocytes are highly plastic cells and critically contribute to physiology and pathophysiology. Monocytes are well established to give rise to tissue macrophages. In parallel these cells are assumed to give to monocyte-derived DC, an assumption that is however largely based on in vitro system but has been difficult to confirm in vivo. Specifically, with the recent understanding of the complexity of classical DC ontogeny, the existence of monocyte-derived DC as a terminally differentiated product of monocytes, rather than an intermediate state, has been challenged.

Here the authors used comprehensive temporal scRNA sequencing (scRNA-seq) of human monocyte cultures and murine engrafted monocytes in a peritonitis model to dissect differentiation trajectories of these cells. In both systems, the authors observe clear and convincing evidence for divergent differentiation paths that could indicate mo-Mac versus mo-DC potential. Moreover, shRNA silencing of selected TF factors selectively impaired the two paths in the human monocyte cultures, corroborating their existence. Specifically, the authors show IRF1 required for mo-Mac differentiation, while ZNF366 and MAFF promoted differentiation they interpret as mo-DC.

Collectively, this well-performed and clearly written study establish the potential of monocytes to give rise to distinct progeny, in line with a model that proposes that mo-Mac and mo-DC derive from alternative differentiation pathways that are controlled by distinct regulatory networks. This in an important study to dissect the differentiation potential of monocytes.

The question of whether mo-DC exists will require a side by side comparison of these cells with emerging cDC1/DC2 and cDC3 precursors, as well as the definition of unique functional features of these cells in physiological context, both of which is beyond the scope of this study.

Specific points:

Given the fact that monocytes and preDC are phenotypically quite similar (Menezes 2016 Immunity), the authors might want to show some preDC markers in their sc experiment, such as the one found in Anderson et al., 2020, Nat Rev Immunol.

The adoptive monocyte experiment is fairly convincing and supports the human data. The evidence for CD11c in the tSNE in Fig2D, is however somehow weak. Can the authors complement this with a dotplot or histogram. Also, it would be even more convincing if the authors could include more markers to establish phenotypic differences between mo-DC and mo-Mac such as CX3CR1, CD64, PDL1, CD209a.

The authors chose to perform the IRF1 knock down in a moMac-specific culture. Was this for technical reasons? How does this manipulation play out in the regular medium of MCSF/IL4 and TNF ?

The Western blot evidence for ZNF366 knock-down is less convincing but the outcome, i.e. reduction moDC makes up for that. Concerning MAFF, the Western blot is more convincing. However, moMac are dramatically reduced in all conditions, representing only 5% of total cells, even in the shControl (Fig 5G). By comparison, in the previous experiment, moMac make up

20% of total cells in the shControl (Fig 5D). This is also the case in Fig4F (control). The authors should explain this discrepancies. Similarly, in Fig4F, moDC make up only 13.3% of total cells in an unperturbed setting whereas in Fig 5, the default in shControl samples is 25-30% of total cells. Please comment.

Referee #1:

This study by Villar et al. examined mo-Mac versus mo-DC differentiation pathway in both human and mouse models. The authors rely on temporal single-cell RNA sequencing followed by RNA velocity analysis using scVelo (human, in vitro) and pseudotime trajectory analysis using Slingshot (mouse, in vivo) to get the conclusion that, mo-Mac and mo-DC develop via two divergent and alternative differentiation pathways. In addition to the author's previous discoveries, this study further identified the transcription factors IRF1, ZNF366 and MAFF to be regulators of mo-Mac and mo-DC differentiation.

While RNA velocity analysis and pseudotime trajectory analysis can suggest potential developmental pathway, these computational methods alone cannot serve as the definitive evidence to prove the "divergent and alternative differentiation pathways". The transcription factors IRF1, ZNF366 and MAFF were only partially required for mo-Mac or mo-DC differentiation, and the effect of IRF1 is tested in a culture condition that only generate mo-Mac but not mo-DC.

Overall, this study does not have strong enough evidence to support the conclusion that "mo-Mac and mo-DC represent two alternative cell fates requiring distinct transcription factors for their differentiation".

We regret that this reviewer was not interested in our work. In the absence of specific comments, we cannot address the concerns of this reviewer.

Referee #2:

The paper by Villar and colleagues tackles the long-lasting question about the differentiation of monocytes into macrophages and dendritic cells. The authors use an in vitro approach to investigate the temporal differentiation of monocytes by scRNA-Seq. Based on RNA velocity analysis, the authors claim to identify a splitting point, in which monocytes either develop into DCs or macrophages. Additionally they profile monocyte-derived cells at different time-points after transfer into the peritoneum of thioglycolate-injected animals by flow cytometry and found a similar bifurcation. The authors extracted three transcription factors that might play a role in this separation, namely Irf1, Znf366 and MAFF. Indeed, silencing each one of them let to reduced macrophage and DC development, respectively.

The experiments are well-performed and the writing is clear. However, there are some important things that need be addressed:

1. For the experiments represented in Fig. 2 the authors isolated monocytes from the BM with an isolation kit. The company of this kit gives no information about the antibodies used to select lineage positive cells nor do the authors show the purity of cells after isolation. Since the three hours UMAP blot in Fig. 2C suggest that maybe 50% of CD45.2+ cells are Ly6C- (corresponding to clusters 2/3/14 in 2G), it is important to know, if this reflects down-regulation or contamination with other BM cells such as precursors or DCs. This is of importance cause these cells could represent an

alternative precursor subset for moDC or macrophages. What happens for instance, when the authors use cluster 2 instead of cluster 12 as starting point for their slingshot analysis? FACS-sorted cells should be used for these kind of experiments.

We have performed flow cytometry analysis of the purified population after isolation with the monocyte kit. We found that the purity of Ly6C+ monocyte was around 80%, while there was only a marginal contamination with CD43+ monocytes or pre-cDC. We added this data in supplementary information (figS2C).

This data suggests that the Ly6C- population present in peritoneum 3 hours after monocyte transfer results from the down-regulation of Ly6C on monocytes.

We have performed the trajectory analysis using this cluster 2 as starting point, and we found similar results, i.e. two divergent pathways leading to moDC or moMac clusters. This analysis is shown below for the reviewer's appreciation, but we believe it will be redundant and unnecessary to add it in the manuscript.

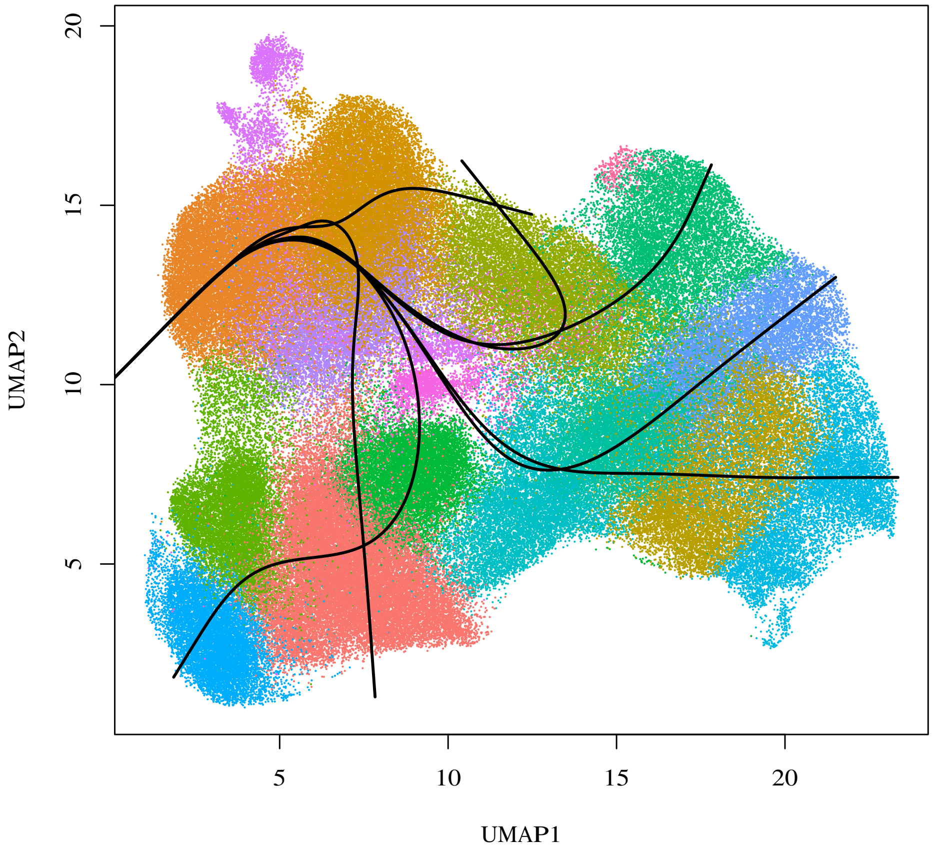

2. It is unclear, how the authors identified IRF1, ZNF366 and MAFF as possible candidates in Fig. 3. What is the logic of the selection? The authors mention: 'we selected for further study IRF1, which is more expressed in macrophage-engaged clusters 4 and 6 and shows high transcriptional activity in the clusters 5 and 6'. Isn't the same true for STAT2, IRF9 and STAT1? Why didn't they choose these genes? MAFF and ZNF366 are also not listed in Fig. 3E and their selection is even not explained. Maybe an analysis using LISA (http://lisa.cistrome.org/) might help to justify their choice.

To address the reviewer's comments, we have added some references to better explain our selection of candidate molecules. "Among differentially expressed transcription factors, we also selected for further study *ZNF366*, which is more expressed in DC-committed clusters 3 (fig3A) and 8 (fig3C) and whose mouse ortolog is involved in classical DC1 (cDC1) terminal differentiation (Chopin et al., 2019; Shengbo et al., 2021). Finally we selected *MAFF*, which is more expressed in macrophage-committed clusters 6 (fig3B) and 14 (fig3D) and heterodimerizes with the transcription factor NFE2L2 (Katsuoka & Yamamoto, 2016) which showed high predicted activity in clusters 5 and 6 (fig3E)."
Regarding STAT1, *STAT1* was also more expressed in cluster 6 (fig3C) with high predicted activity in clusters 5 and 6 (fig3E). However, STAT1 phosphorylation, which is required for its transcriptional activity, was almost undetectable (see figure below), therefore we did not select STAT1 for further study. We believe it is not necessary to add this data to the manuscript.

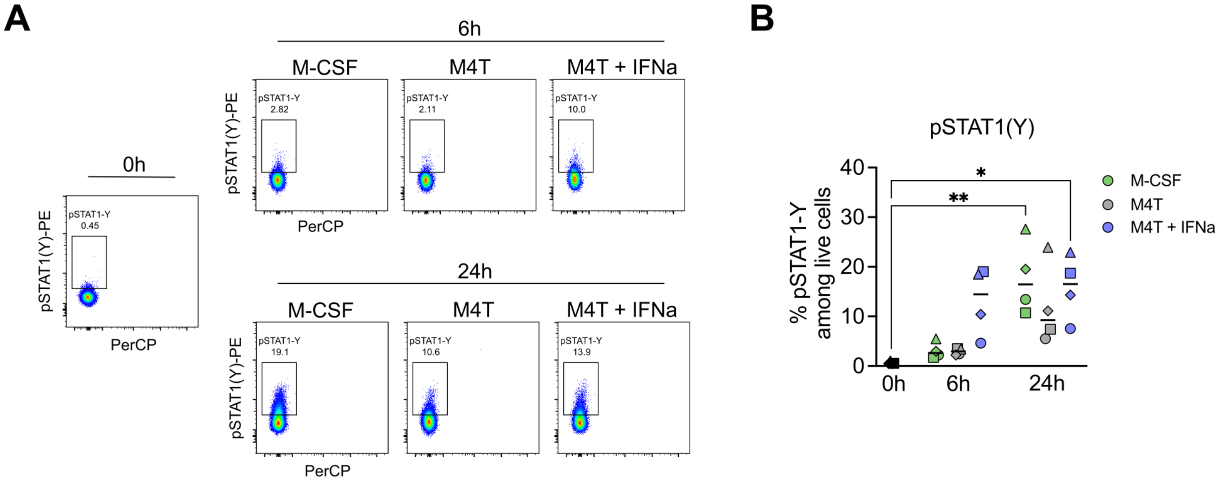

Monocytes were cultured with MCSF or MCSF+IL4+TNFa, with or without IFNa as a positive control. STAT1 phosphorylation was assessed by intra-cellular flow cytometry after 6h or 24h of culture. (A) Representative results. (B) Quantification (n=4 in 2 independent experiments.

3. The authors show in Fig. 4B that IRF1 influences macrophage development by reducing the frequency of CD16+ cells two-fold. Later experiments in this figure suggest that the expression of ISG genes does not influence moDC development. Accordingly, the authors should additionally show, how IRF1 silencing influences the differentiation of cells under M4T conditions. A cell quantification rather than a frequency analysis should be performed to show that moDC development is unaffected by IRF1 silencing in M4T-treated cells. The authors should also add IRF1 expression to the real time PCR quantification shown in Fig. 4E to examine the expression changes based on culture condition.
We have now added the silencing experiment for IRF1 in the culture with MCSF, IL4 and TNFa. We found that moMac differentiation was significantly decreased upon silencing, but moDC were unaffected (fig 4D).
We have also performed an analysis of IRF1 protein expression (by Western Blot) at different time points, including day 0 (= freshly isolated monocytes). We found that IRF1 is already expressed in monocytes (fig4A), and its expression increases after 1 day in culture.

4. The mechanism, how IRF1, ZNF366 and MAFF influence the development of monocytes is completely unclear. Maybe a molecular analysis is too much to ask for, even though feasible, but at least the survival and apoptosis rate of monocyte-derived cells after gene silencing at different time points should be analysed. In all our silencing experiments, cell viability was similar between conditions. Unfortunately, we did not count the absolute number of cells recovered at the end point. During revisions, we performed volumetric counting for the IRF1 silencing experiment, and we did not find significant differences in the number of cells recovered for each condition (see figure below). Therefore, we believe that showing the percentage of each population provides the same information as the absolute number of recovered cells. Understanding how IRF1, ZNF366 or MAFF impact monocyte differentiation is beyond the scope of the present manuscript and could represent entire projects on their own.

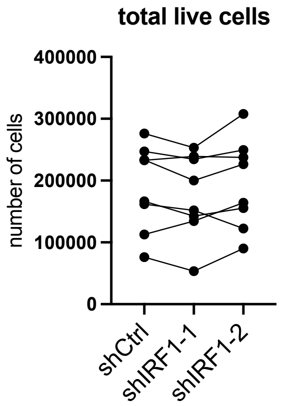

Each dot represents an individual donor (n=9 in 3 independent experiments).

Minor things:

-The current title emphasises that the here identified transcriptional program is conserved in all monocyte to macrophage or DC transition scenarios. This, however, seems to be a generalisation. As mentioned by the authors themselves, there finding might not be true during neuroinflammation. Accordingly, the title should be adjusted to fit the actual findings (which is under in vitro conditions and during peritonitis) We agree with the reviewer that different trajectories may exist in different inflammatory contexts. Indeed, we discuss this point in the discussion section, and we have been careful not to overstate our conclusions. In addition, we have clearly indicated the experimental conditions we have used in the abstract of the manuscript : “in an *in vitro* model” and “a mouse model of peritonitis”. We respectfully disagree with the reviewer concerning the necessity to modify the title. Titles need to be short and cannot convey all the subtleties of a paper.

-In respect to Fig. 1 the authors write on page 5: 'these results reveal two divergent and alternative differentiation pathways'. This statement is solely based on RNA velocity analysis. However, RNA velocity is an algorithm that relies on the assumption that a molecular connection between cells really exists. Time point analysis of RBC differentiation showed that the RNA velocity alone is not able to predict an experimental induced differentiation path, at least not with adjustments (Barile et al., 2021). Therefore RNA velocity cannot replace experimental proof and is just a possible interpretation of biological findings. Accordingly it should not be stated that 'these results reveal (...) differentiation' but instead 'these results suggest (...) differentiation'.

We agree with the reviewer and the sentence has been modified according to the reviewer's suggestion.

-Could the authors add IRF1, MAFF and ZNF366 to the UMAP represented in Fig. 1D or to later figures? A time-dependent validation of gene expression with real-time PCR should be added as well.
We have now added an analysis of protein expression for these molecules at different time points, including day 0 (= freshly isolated monocytes), for IRF1, ZNF366 and MAFF. We found that IRF1 is expressed in monocytes (fig4A), while ZNF366 and MAFF are expressed only upon differentiation (fig5C and fig5G).

-Also the expression of other small MAF family members should be represented as UMAPs.
Below is the representation of MAF molecules expression for the reviewer's appreciation. We do not believe that it is relevant to show it in the manuscript, but we could add this data in the supplementary information if the editor thinks it is necessary.

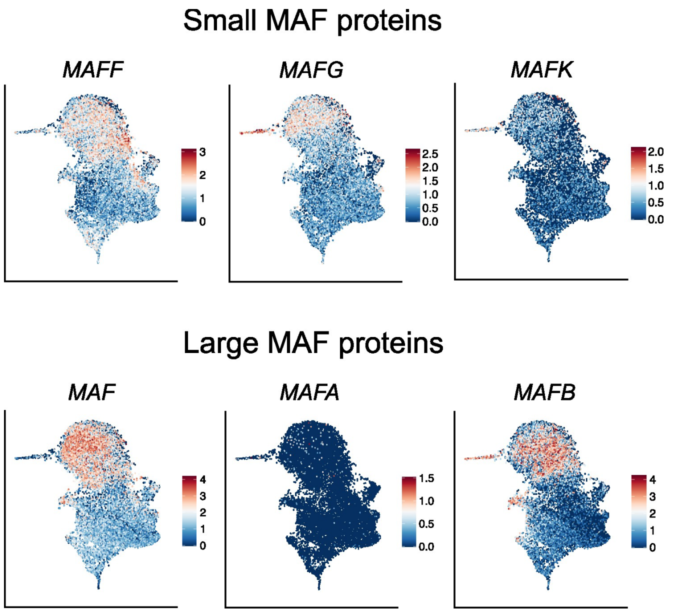

-The authors write in figure legend 5D that the "Median is shown (n=8 in 3 independent experiments)", but the blot shows five data points. The data points mentioned in figure legend 5G also do not fit to the data shown in the figure. Can the authors clarify this point.
Thank you for pointing this out. It has been corrected.

-It is not completely clear, if 5-days cultured monocytes were used for Immunoblot analysis shown in Fig. 5C and 5F. Are ZNF366 and MAFF expressed under steady state conditions? An Immunoblot analysis of freshly isolated monocytes should be added.
These Western blots were performed after 5 days of culture, as indicated in the legend. We have now added an additional Western Blot analysis at different time points, including day 0 (= freshly isolated monocytes), for IRF1, ZNF366 and MAFF. We found that IRF1 is expressed in monocytes (fig4A), while ZNF366 and MAFF are expressed only upon differentiation (fig5C and fig5G).

Referee #3:

Monocytes are highly plastic cells and critically contribute to physiology and pathophysiology. Monocytes are well established to give rise to tissue macrophages. In parallel these cells are assumed to give to monocyte-derived DC, an assumption that is however largely based on in vitro system but has been difficult to confirm in vivo. Specifically, with the recent understanding of the complexity of classical DC ontogeny, the existence of monocyte-derived DC as a terminally differentiated product of monocytes, rather than an intermediate state, has been challenged.

Here the authors used comprehensive temporal scRNA sequencing (scRNA-seq) of human monocyte cultures and murine engrafted monocytes in a peritonitis model to dissect differentiation trajectories of these cells. In both systems, the authors observe clear and convincing evidence for divergent differentiation paths that could indicate mo-Mac versus mo-DC potential. Moreover, shRNA silencing of selected TF factors selectively impaired the two paths in the human monocyte cultures, corroborating their existence. Specifically, the authors show IRF1 required for mo-Mac differentiation, while ZNF366 and MAFF promoted differentiation they interpret as mo-DC.

Collectively, this well-performed and clearly written study establish the potential of monocytes to give rise to distinct progeny, in line with a model that proposes that mo-Mac and mo-DC derive from alternative differentiation pathways that are controlled by distinct regulatory networks. This in an important study to dissect the differentiation potential of monocytes.

The question of whether mo-DC exists will require a side by side comparison of these cells with emerging cDC1/DC2 and cDC3 precursors, as well as the definition of unique functional features of these cells in physiological context, both of which is beyond the scope of this study.

Specific points:

Given the fact that monocytes and preDC are phenotypically quite similar (Menezes 2016 Immunity), the authors might want to show some preDC markers in their sc experiment, such as the one found in Anderson et al., 2020, Nat Rev Immunol.
This is an important point. In a recent publication, we have performed scRNA-seq on the isolated monocytes used in these cultures, and shown that they do not contain contaminating DC precursors (Villar et al., 2022). We have added a reference to this publication in the text.

The adoptive monocyte experiment is fairly convincing and supports the human data. The evidence for CD11c in the tSNE in Fig2D, is however somehow weak. Can the authors complement this with a dotplot or histogram. Also, it would be even more convincing if the authors could include more markers to establish phenotypic differences between mo-DC and mo-Mac such as CX3CR1, CD64, PDL1, CD209a.
We have previously characterized the morphology and phenotype of peritoneal moDC (Goudot et al., 2017). To complement this, we have characterized the phenotype of moDC and moMac during peritonitis. We found that CD11c was expressed only on

moDC while moMac had higher expression of F4/80, CD64 and MerTK. We have added this data to figure S2. By contrast, the expression of PDL1 or CD209a was not different between moDC and moMac (not shown).

The authors chose to perform the IRF1 knock down in a moMac-specific culture. Was this for technical reasons? How does this manipulation play out in the regular medium of MCSF/IL4 and TNF ?
We have now added the silencing experiment for IRF1 in the culture with MCSF, IL4 and TNFa. We found that moMac differentiation was significantly decreased upon silencing, but moDC were unaffected (fig 4D).

The Western blot evidence for ZNF366 knock-down is less convincing but the outcome, i.e. reduction moDC makes up for that.
Concerning MAFF, the Western blot is more convincing. However, moMac are dramatically reduced in all conditions, representing only 5% of total cells, even in the shControl (Fig 5G). By comparison, in the previous experiment, moMac make up 20% of total cells in the shControl (Fig 5D). This is also the case in Fig4F (control). The authors should explain this discrepancies. Similarly, in Fig4F, moDC make up only 13.3% of total cells in an unperturbed setting whereas in Fig 5, the default in shControl samples is 25-30% of total cells. Please comment.
These differences in the percentage of moDC and moMac represent inter-individual variations. We have already observed a similar range of variation in our previous work in which we characterized this culture system (Coillard et al., 2021; Goudot et al., 2017). This is inherent to this system based on human cells, as monocytes can be influenced in blood donors by many environmental factors that we cannot control, including circadian rhythm, diet, gender, age (Patel & Yona, 2019). To acknowledge this variability, we represent symbols of individual donors in our figures. To ensure the robustness of our findings, we used multiple individual donors in each experiment. Importantly, the effect of each silencing is reproducible and consistent between donors independently of the proportion of each population in the control condition. Moreover, we performed paired statistical analysis, and the results were significant in each case.

References

Chopin, M., Lun, A. T., Zhan, Y., Schreuder, J., Coughlan, H., D’Amico, A., Mielke, L. A., Almeida, F. F., Kueh, A. J., Dickins, R. A., Belz, G. T., Naik, S. H., Lew, A. M., Bouillet, P., Herold, M. J., Smyth, G. K., Corcoran, L. M., & Nutt, S. L. (2019). Transcription Factor PU.1 Promotes Conventional Dendritic Cell Identity and Function via Induction of Transcriptional Regulator DC-SCRIPT. *Immunity*, *50*(1), 77-90.e5. https://doi.org/https://doi.org/10.1016/j.immuni.2018.11.010

Coillard, A., Guyonnet, L., De Juan, A., Cros, A., & Segura, E. (2021). TLR or NOD receptor signaling skews monocyte fate decision via distinct mechanisms driven by mTOR and miR-155. *Proceedings of the National Academy of Sciences*, *118*(43), e2109225118. https://doi.org/10.1073/pnas.2109225118

Goudot, C., Coillard, A., Villani, A. C., Gueguen, P., Cros, A., Sarkizova, S., Tang-Huau, T. L., Bohec, M., Baulande, S., Hacohen, N., Amigorena, S., & Segura, E. (2017). Aryl Hydrocarbon Receptor Controls Monocyte Differentiation into Dendritic Cells versus Macrophages. *Immunity*, *47*(3), 582-596.e6. https://doi.org/10.1016/J.IMMUNI.2017.08.016

Katsuoka, F., & Yamamoto, M. (2016). Small Maf proteins (MafF, MafG, MafK): History, structure and function. *Gene*, *586*(2), 197–205. https://doi.org/10.1016/j.gene.2016.03.058

Patel, A. A., & Yona, S. (2019). Inherited and Environmental Factors Influence Human Monocyte Heterogeneity. *Frontiers in Immunology*, *10*, 2581. https://doi.org/10.3389/FIMMU.2019.02581/BIBTEX

Shengbo, Z., D., C. H., Mitra, A., Simona, S., J., K. A., V., B. D., Wang, C., Nicolas, J., Angela, D., M., L. A., Yifan, Z., J., T. C., A., V. J., K., S. G., Michaël, C., & L., N. S. (2021). Type 1 conventional dendritic cell fate and function are controlled by DC-SCRIPT. *Science Immunology*, *6*(58), eabf4432. https://doi.org/10.1126/sciimmunol.abf4432

Villar, J., Cros, A., De Juan, A., Alaoui, L., Bonte, P. E., Lau, C. M., Tiniakou, I., Reizis, B., & Segura, E. (2022). ETV3 and ETV6 enable monocyte differentiation into dendritic cells by repressing macrophage fate commitment. *Nature Immunology 2022 24:1*, *24*(1), 84–95. https://doi.org/10.1038/s41590-022-01374-0

Dear Dr. Segura,

Thank you for the submission of your revised manuscript to our editorial offices. I have now received the reports from the two referees that I asked to re-evaluate the study, you will find below. As you will see, both referees now fully support the publication of your paper in EMBO reports. Referee #2 has some suggestions to improve the study I ask you to address in a final revised manuscript. Please also provide a brief final p-b-p-response addressing these points.

Moreover, I have these editorial requests I also ask you to address:

- Please provide a shorter and comprehensive title with not more than 100 characters (including spaces).

- Please provide the abstract written in present tense throughout.

- We plan to publish your manuscript in the Report format (as you also indicated in the submission system). For a Scientific Report we require that results and discussion sections are combined in a single chapter called "Results & Discussion". Please do this for your manuscript. For more details, please refer to our guide to authors:
http://www.embopress.org/page/journal/14693178/authorguide#researcharticleguide

- We now use CRediT to specify the contributions of each author in the journal submission system. CRediT replaces the author contribution section. Please use the free text box to provide more detailed descriptions. Thus, please remove the author contributions section from the manuscript text file. See also guide to authors:
https://www.embopress.org/page/journal/14693178/authorguide#authorshipguidelines

- Regarding data quantification and statistics, please make sure that the number "n" for how many independent experiments were performed, their nature (biological versus technical replicates), the bars and error bars (e.g. SEM, SD) and the test used to calculate p-values is indicated in the respective figure legends (also for potential EV figures and all those in the final Appendix). Please also check that all the p-values are explained in the legend, and that these fit to those shown in the figure. Please provide statistical testing where applicable. Please avoid the phrase 'independent experiment', but clearly state if these were biological or technical replicates. Please also indicate (e.g. with n.s.) if testing was performed, but the differences are not significant. In case n=2, please show the data as separate datapoints or bars without error bars and statistics. See also:
http://www.embopress.org/page/journal/14693178/authorguide#statisticalanalysis

If n<5, please show single datapoints for diagrams. Presently, it seems there are diagrams with no or only partial statistics (e.g. in panels 4D, 4G, 4H, 5D, 5E, 5I and EV2c). Please check.

- Please make sure that all the funding information is also entered into the online submission system and that it is complete and similar to the one in the acknowledgement section of the manuscript text file.

- Please add a direct link to reach the GEO dataset GSE218483 to the data availability section.

- As I already indicated in my first decision letter, we now request the publication of original source data with the aim of making primary data more accessible and transparent to the reader. It seems you have been contacted already by our source data coordinator, who indicated which figure panels we would need source data for. I attach again the source data checklist and the FAQ. Please make sure that all the requested source data is provided. Please include here also the source data for all Western blots (see the comment by referee #2 - i.e. uncropped original versions with size markers). Please upload all source data for one figure as one pdf per figure or (if there is more than one file) ZIPed together as one folder. Finally, please upload the filled in source data checklist with your final revised files.

- The two Appendix Tables are datasets. Please upload these as dataset excel files (named Dataset EV1 and Dataset EV2) with a title and a legend on the first TAB. Finally, please change the callouts for these files.

- Please use our reference format (we need et al if there are more then10 authors, please remove the DOIs and add the issue and page numbers):
http://www.embopress.org/page/journal/14693178/authorguide#referencesformat

- Finally, please find attached a word file of the manuscript text (provided by our publisher) with changes we ask you to include in your final manuscript text and comments. Please use the attached file as basis for further revisions and provide your final manuscript file with track changes, in order that we can see any modifications done.

In addition, I would need from you:
- a short, two-sentence summary of the manuscript (not more than 35 words).
- two to four short bullet points highlighting the key findings of your study (two lines each).

- a schematic summary figure that provides a sketch of the major findings (not a data image) in jpeg or tiff format (with the exact width of 550 pixels and a height of not more than 400 pixels) that can be used as a visual synopsis on our website.

I look forward to seeing the final revised version of your manuscript when it is ready. Please let me know if you have questions regarding the revision.

Please use this link to submit your revision: https://embor.msubmit.net/cgi-bin/main.plex

Best,

Achim Breiling
Senior Editor
EMBO Reports

Referee #2:

The authors answered all my questions sufficiently. However, I still believe that the title of the manuscript is an overstatement and should be adjusted to the actual findings, as mentioned before. But I leave this decision to the editor.

Some minor things:
-Please show the complete Western Blots in the supplement.
-Can the authors show less cells for Suppl. Fig. 2B (middle FACS blot)? Currently it looks like a lot of CD45.1+ CD45.2+ double positive cells. Maybe also doublet removal should help. This might also solve the issue that some CD45.2+ cells are Timd4 and Ly6G positive (right blot), which should not be the case after the transfer of a pure population of monocytes.

Referee #3:

The authors have responded to all the points we raised with satisfaction.

Reply to reviewers – revised manuscript

The authors answered all my questions sufficiently. However, I still believe that the title of the manuscript is an overstatement and should be adjusted to the actual findings, as mentioned before. But I leave this decision to the editor.

To address the reviewer' comments, and to comply with EMBO Reports' 100-characters limit, we have modified the title to 'Monocytes differentiate along two alternative pathways during sterile inflammation'. We believe that the term 'sterile inflammation' adequately reflects the fact that our findings may not be generalizable to all inflammatory contexts.

Some minor things:
-Please show the complete Western Blots in the supplement.
Source data has been added.

-Can the authors show less cells for Suppl. Fig. 2B (middle FACS blot)? Currently it looks like a lot of CD45.1+ CD45.2+ double positive cells. Maybe also doublet removal should help. This might also solve the issue that some CD45.2+ cells are Timd4 and Ly6G positive (right blot), which should not be the case after the transfer of a pure population of monocytes.

We had already excluded doublets in our analysis. See below the complete gating strategy for the reviewer's appreciation. Following the reviewer' suggestion, we have replace the dot plots in the figure with contour plots, better depicting the main populations.

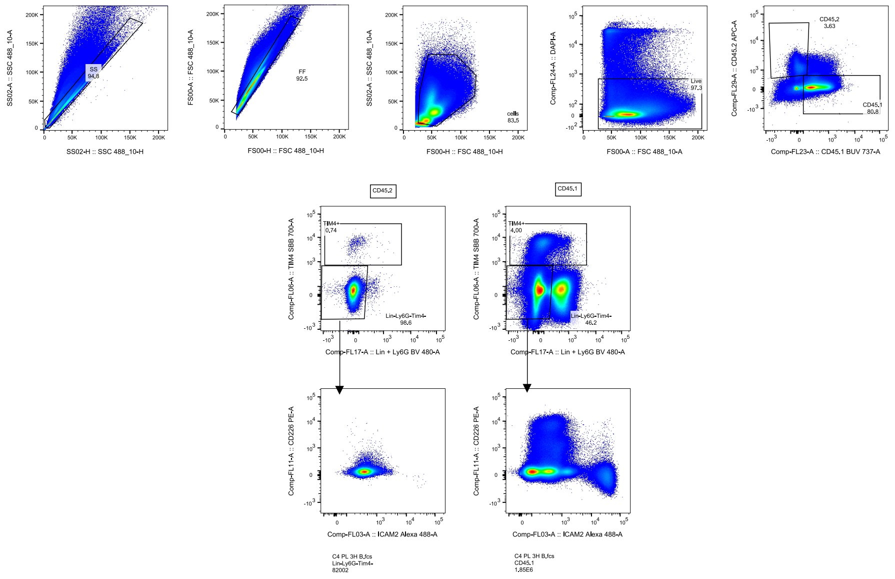

Elodie Segura
Institut Curie
INSERM U932
Paris 75005
France

Dear Dr. Segura,

I am very pleased to accept your manuscript for publication in the next available issue of EMBO reports. Thank you for your contribution to our journal.

At the end of this email I include important information about how to proceed. Please ensure that you take the time to read the information and complete and return the necessary forms to allow us to publish your manuscript as quickly as possible.

As part of the EMBO publication's Transparent Editorial Process, EMBO reports publishes online a Review Process File to accompany accepted manuscripts. As you are aware, this File will be published in conjunction with your paper and will include the referee reports, your point-by-point response and all pertinent correspondence relating to the manuscript.

If you do NOT want this File to be published, please inform the editorial office within 2 days, if you have not done so already, otherwise the File will be published by default [contact: emboreports@embo.org]. If you do opt out, the Review Process File link will point to the following statement: "No Review Process File is available with this article, as the authors have chosen not to make the review process public in this case."

Should you be planning a Press Release on your article, please get in contact with emboreports@wiley.com as early as possible, in order to coordinate publication and release dates.

Thank you again for your contribution to EMBO reports and congratulations on a successful publication. Please consider us again in the future for your most exciting work.

Yours sincerely,

Achim Breiling
Senior Editor
EMBO Reports

****

THINGS TO DO NOW:

Please note that you will be contacted by Wiley Author Services to complete licensing and payment information. The required 'Page Charges Authorization Form' is available here: https://www.embopress.org/pb-assets/embo-site/er_apc.pdf - please download and complete the form and return to embopressproduction@wiley.com

EMBO Press participates in many Publish and Read agreements that allow authors to publish Open Access with reduced/no publication charges. Check your eligibility: https://authorservices.wiley.com/author-resources/Journal-Authors/open-access/affiliation-policies-payments/index.html

You will receive proofs by e-mail approximately 2-3 weeks after all relevant files have been sent to our Production Office; you should return your corrections within 2 days of receiving the proofs.

Please inform us if there is likely to be any difficulty in reaching you at the above address at that time. Failure to meet our deadlines may result in a delay of publication, or publication without your corrections.

All further communications concerning your paper should quote reference number EMBOR-2022-56308V3 and be addressed to emboreports@wiley.com.

Should you be planning a Press Release on your article, please get in contact with emboreports@wiley.com as early as possible, in order to coordinate publication and release dates.

EMBO Press Author Checklist

Corresponding Author Name: Elodie Segura	
Journal Submitted to: EMBO Reports	
Manuscript Number: EMBOR-2022-56308V2	

USEFUL LINKS FOR COMPLETING THIS FORM
The EMBO Journal - Author Guidelines
EMBO Reports - Author Guidelines
Molecular Systems Biology - Author Guidelines
EMBO Molecular Medicine - Author Guidelines

Reporting Checklist for Life Science Articles (updated January 2022)
This checklist is adapted from Materials Design Analysis Reporting (MDAR) Checklist for Authors. MDAR establishes a minimum set of requirements in transparent reporting in the life sciences (see Statement of Task: 10.31222/osf.io/9sm4x). Please follow the journal's guidelines in preparing your manuscript.
Please note that a copy of this checklist will be published alongside your article.

Abridged guidelines for figures
1. Data
The data shown in figures should satisfy the following conditions:
- the data were obtained and processed according to the field's best practice and are presented to reflect the results of the experiments in an accurate and unbiased manner.
- ideally, figure panels should include only measurements that are directly comparable to each other and obtained with the same assay.
- plots include clearly labeled error bars for independent experiments and sample sizes. Unless justified, error bars should not be shown for technical replicates.
- if n<5, the individual data points from each experiment should be plotted. Any statistical test employed should be justified.
- Source Data should be included to report the data underlying figures according to the guidelines set out in the authorship guidelines on Data Presentation.

2. Captions
Each figure caption should contain the following information, for each panel where they are relevant:
- a specification of the experimental system investigated (eg cell line, species name).
- the assay(s) and method(s) used to carry out the reported observations and measurements.
- an explicit mention of the biological and chemical entity(ies) that are being measured.
- an explicit mention of the biological and chemical entity(ies) that are altered/varied/perturbed in a controlled manner.
- the exact sample size (n) for each experimental group/condition, given as a number, not a range;
- a description of the sample collection allowing the reader to understand whether the samples represent technical or biological replicates (including how many animals, litters, cultures, etc.).
- a statement of how many times the experiment shown was independently replicated in the laboratory.
- definitions of statistical methods and measures:
  - common tests, such as t-test (please specify whether paired vs. unpaired), simple χ2 tests, Wilcoxon and Mann-Whitney tests, can be unambiguously identified by name only, but more complex techniques should be described in the methods section;
  - are tests one-sided or two-sided?
  - are there adjustments for multiple comparisons?
  - exact statistical test results, e.g., P values = x but not P values < x;
  - definition of 'center values' as median or average;
  - definition of error bars as s.d. or s.e.m.

Please complete ALL of the questions below.
Select "Not Applicable" only when the requested information is not relevant for your study.

Materials

Newly Created Materials	Information included in the manuscript?	In which section is the information available? (Reagents and Tools Table, Materials and Methods, Figures, Data Availability Section)
New materials and reagents need to be available; do any restrictions apply?	Not Applicable	

Antibodies	Information included in the manuscript?	In which section is the information available? (Reagents and Tools Table, Materials and Methods, Figures, Data Availability Section)
For **antibodies** provide the following information:<br>- Commercial antibodies: RRID (if possible) or supplier name, catalogue number and or/clone number<br>- Non-commercial: RRID or citation	Yes	Material and Methods

DNA and RNA sequences	Information included in the manuscript?	In which section is the information available? (Reagents and Tools Table, Materials and Methods, Figures, Data Availability Section)
**Short novel DNA or RNA including primers, probes**: provide the sequences.	Not Applicable	

Cell materials	Information included in the manuscript?	In which section is the information available? (Reagents and Tools Table, Materials and Methods, Figures, Data Availability Section)
**Cell lines:** Provide species information, strain. Provide accession number in repository **OR** supplier name, catalog number, clone number, and/**OR** RRID.	Not Applicable	
**Primary cultures:** Provide species, strain, sex of origin, genetic modification status.	Yes	Material and Methods
Report if the cell lines were recently **authenticated** (e.g., by STR profiling) and tested for mycoplasma contamination.	Not Applicable	

Experimental animals	Information included in the manuscript?	In which section is the information available? (Reagents and Tools Table, Materials and Methods, Figures, Data Availability Section)
**Laboratory animals or Model organisms:** Provide species, strain, sex, age, genetic modification status. Provide accession number in repository **OR** supplier name, catalog number, clone number, **OR** RRID.	Yes	Material and Methods
**Animal observed in or captured from the field:** Provide species, sex, and age where possible.	Not Applicable	
Please detail **housing and husbandry conditions**.	Yes	Material and Methods

Plants and microbes	Information included in the manuscript?	In which section is the information available? (Reagents and Tools Table, Materials and Methods, Figures, Data Availability Section)
**Plants:** provide species and strain, ecotype and cultivar where relevant, unique accession number if available, and source (including location for collected wild specimens).	Not Applicable	
**Microbes:** provide species and strain, unique accession number if available, and source.	Not Applicable	

Human research participants	Information included in the manuscript?	In which section is the information available? (Reagents and Tools Table, Materials and Methods, Figures, Data Availability Section)
If collected and within the bounds of privacy constraints report on age, sex and gender or ethnicity for all study participants.	Not Applicable	

Core facilities	Information included in the manuscript?	In which section is the information available? (Reagents and Tools Table, Materials and Methods, Figures, Data Availability Section)
If your work benefited from core facilities, was their service mentioned in the acknowledgments section?	Yes	Acknowledgements

Design

Study protocol	Information included in the manuscript?	In which section is the information available? (Reagents and Tools Table, Materials and Methods, Figures, Data Availability Section)
If study protocol has been **pre-registered, provide DOI in the manuscript**. For clinical trials, provide the trial registration number **OR** cite DOI.	Not Applicable	
Report the **clinical trial registration number** (at ClinicalTrials.gov or equivalent), where applicable.	Not Applicable	

Laboratory protocol	Information included in the manuscript?	In which section is the information available? (Reagents and Tools Table, Materials and Methods, Figures, Data Availability Section)
Provide DOI OR other citation details if **external detailed step-by-step protocols** are available.	Not Applicable	

Experimental study design and statistics	Information included in the manuscript?	In which section is the information available? (Reagents and Tools Table, Materials and Methods, Figures, Data Availability Section)
Include a statement about **sample size** estimate even if no statistical methods were used.	Yes	Material and methods
Were any steps taken to minimize the effects of subjective bias when allocating animals/samples to treatment (e.g. **randomization procedure**)? If yes, have they been described?	Not Applicable	
Include a statement about **blinding** even if no blinding was done.	Yes	Material and methods
Describe **inclusion/exclusion criteria** if samples or animals were excluded from the analysis. Were the criteria pre-established?<br><br>If sample or data points were omitted from analysis, report if this was due to attrition or intentional exclusion and provide justification.	Yes	Material and methods
For every figure, are **statistical tests** justified as appropriate? Do the data meet the assumptions of the tests (e.g., normal distribution)? Describe any methods used to assess it. Is there an estimate of variation within each group of data? Is the variance similar between the groups that are being statistically compared?	Yes	

Sample definition and in-laboratory replication	Information included in the manuscript?	In which section is the information available? (Reagents and Tools Table, Materials and Methods, Figures, Data Availability Section)
In the figure legends: state number of times the experiment was **replicated** in laboratory.	Yes	
In the figure legends: define whether data describe **technical or biological replicates**.	Yes	

Ethics

Ethics	Information included in the manuscript?	In which section is the information available? (Reagents and Tools Table, Materials and Methods, Figures, Data Availability Section)
Studies involving **human participants**: State details of **authority granting ethics approval** (IRB or equivalent committee(s), provide reference number for approval.	Yes	Material and methods
Studies involving **human participants**: Include a statement confirming that **informed consent** was obtained from all subjects and that the experiments conformed to the principles set out in the WMA Declaration of Helsinki and the Department of Health and Human Services Belmont Report.	Not Applicable	
Studies involving **human participants:** For publication of **patient photos**, include a statement confirming that consent to publish was obtained.	Not Applicable	
Studies involving experimental **animals**: State details of **authority granting ethics approva**l (IRB or equivalent committee(s), provide reference number for approval. Include a statement of compliance with ethical regulations.	Yes	Material and methods
Studies involving **specimen and field samples:** State if relevant **permits** obtained, provide details of authority approving study; if none were required, explain why.	Not Applicable	

Dual Use Research of Concern (DURC)	Information included in the manuscript?	In which section is the information available? (Reagents and Tools Table, Materials and Methods, Figures, Data Availability Section)
Could your study fall under dual use research restrictions? Please check biosecurity documents and list of **select agents and toxins** (CDC): https://www.selectagents.gov/sat/list.htm	Not Applicable	
If you used a select agent, is the security level of the lab appropriate and reported in the manuscript?	Not Applicable	
If a study is subject to dual use research of concern regulations, is the name of the **authority granting approval and reference number** for the regulatory approval provided in the manuscript?	Not Applicable	

Reporting

The MDAR framework recommends adoption of discipline-specific guidelines, established and endorsed through community initiatives. Journals have their own policy about requiring specific guidelines and recommendations to complement MDAR.

Adherence to community standards	Information included in the manuscript?	In which section is the information available? (Reagents and Tools Table, Materials and Methods, Figures, Data Availability Section)
State if relevant guidelines or checklists (e.g., **ICMJE, MIBBI, ARRIVE, PRISMA**) have been followed or provided.	Not Applicable	
For **tumor marker prognostic studies,** we recommend that you follow the **REMARK** reporting guidelines (see link list at top right). See author guidelines, under 'Reporting Guidelines'. Please confirm you have followed these guidelines.	Not Applicable	
For **phase II and III randomized controlled trials**, please refer to the **CONSORT** flow diagram (see link list at top right) and submit the CONSORT checklist (see link list at top right) with your submission. See author guidelines, under 'Reporting Guidelines'. Please confirm you have submitted this list.	Not Applicable	

Data Availability

Data availability	Information included in the manuscript?	In which section is the information available? (Reagents and Tools Table, Materials and Methods, Figures, Data Availability Section)
Have **primary datasets** been deposited according to the journal's guidelines (see 'Data Deposition' section) and the respective accession numbers provided in the Data Availability Section?	Yes	Data availibility statement
Were **human clinical and genomic datasets** deposited in a public access-controlled repository in accordance to ethical obligations to the patients and to the applicable consent agreement?	Not Applicable	
Are **computational models** that are central and integral to a study available without restrictions in a machine-readable form? Were the relevant accession numbers or links provided?	Not Applicable	
If publicly available data were reused, provide the respective **data citations in the reference list**.	Not Applicable	
